# CXC chemokines: Potential biomarker and immunotherapeutic target for uterine corpus endometrial carcinoma

**DOI:** 10.1371/journal.pone.0277872

**Published:** 2024-01-17

**Authors:** Guang Wang, Juan Fu, Mulin Liu, Qin Zheng

**Affiliations:** 1 Department of Dalian Key Laboratory of Reproduction and Mother-child Genetic, Reproductive & Genetic Medicine Center, Dalian Women and Children’s Medical Group, Dalian, Liaoning Province, China; 2 Department of Obstetrics and Gynecology, the First Affiliated Hospital of Dalian Medical University, Dalian, Liaoning Province, China; 3 Department of Clinical Laboratory, the First Affiliated Hospital of Dalian Medical University, Dalian, Liaoning Province, China; 4 Department of Biochemistry and Molecular Biology, Dalian Medical University, Dalian, Liaoning Province, China; Hamad Medical Corporation, QATAR

## Abstract

Uterine corpus endometrial carcinoma (UCEC) is one of the most common type of gynecological malignancies. Multiple lines of evidence indicated that CXC chemokines exerted an anti-tumor immunological role in the tumor microenvironment which were critical regulators of cancer immunity. However, the relevance of CXC chemokines in the evaluation of prognosis and immune infiltration of UCEC remains to be explored. This study utilized various online databases, including TCGA, UALCAN, Kaplan-Meier Plotter, cBioPortal, TIMER2.0, TISIDB, and MethSurv to perform the analysis. Gene expression data from the TCGA-UCEC dataset indicated decreased expression of CXCL2/12 and increased expression of CXCL14/17. CXCL2/12 expression was negatively whereas CXCL14/17 expression was positively correlated with clinicopathological features of UCEC patients, including cancer stage, patients’ age, weight and menopause status. Patients with higher CXCL12/14 expression corresponded with better clinical outcomes, which were not influenced by the genetic alterations. The differential expression of CXCL2/12/14/17 was not only significantly correlated with immune infiltration levels, but also the abundance of immune checkpoint inhibitors. Heatmaps of DNA methylation of CXCL2/12/14/17 were investigated, and 4 CpGs of CXCL2, 16 CpGs of CXCL12, 3 CpGs of CXCL14/17 were identified where altered methylation affected the prognosis of UCEC patients. These findings provided novel insights into the immunologic features of UCEC and might pave the way toward the prognostic evaluation and immunotherapy selection based on CXCL2/12/14/17 expression status.

## Introduction

Uterine corpus endometrial carcinoma (UCEC) is one of the most common prevalent gynecological malignancies which develop from the inner lining cells of the uterus. Despite the available therapy, the disease causes significant morbidity and mortality [1]. The clinical presentation is highly heterogeneous, with the disease frequently occurring in postmenopausal women. It is characterized by varied clinical and pathological features, including postmenopausal or perimenopausal vaginal bleeding, enlarged uterus, pelvic cramping, and abdominal pain, etc [2]. Although the 5-year survival rate of UCEC patients after early diagnosis approaches 90% [3], in patients presenting with aggressive high-grade UCEC is reduced to 20–40%, despite the early detection and diverse treatments have been improved. This poor outcome is partially due to the lack of accurate biomarkers that could identify patients with a high probability of metastasis formation and recurrence [4]. Therefore, a deeper insight into the mechanism involved in the pathogenesis of UCEC holds the promise of identifying potential novel diagnostic and prognostic strategies to improve patients’ survival.

The initiation and progression of cancer is a complex process that involves the interactions between cancer cells, the microenvironment and the immune system. Tumor microenvironment, containing immune cells, mesenchymal cells, endothelial cells, extracellular matrix molecules and inflammatory mediators, plays an essential role in supporting cancer progression [5–7]. Tumor-associated chronic inflammation involves in the recruitment of immune cells to tumor sites, including macrophages and lymphocytes, etc [8–10]. They play critical roles in the initiation, progression, metastasis, and therapeutic responses of cancers. Generally, a more pronounced tumor-infiltrating lymphocytes indicates a better immune reaction that may eliminate cancer cells, and is usually associated with better prognosis and survival [11–13].

Chemokines, chemotactic cytokines or ligands, comprise a large group of low-molecular weight secreted proteins which playing a central role in the activation and recruitment of immune cells to the infected or injured sites, and lead to cancer progression, invasion or metastasis [14–16]. Based on the position of conserved cysteine residues, chemokines can be classified into four subfamilies: C, CXC, CC, and CX3C. Apart from their role in controlling infections, chemokines also influence the extent of tumor infiltration by immune cells, further affecting the tumor immunity [17, 18]. To date, 48 chemokines and 18 chemokine receptors have been identified in humans [19]. Of these, members of the CXC chemokine superfamily are commonly secreted by immune cells or stromal cells in the tumor microenvironment, which can modulate tumor immunity and tumor immunological or biological phenotypes [20–22]. Although the aberrant expression of CXC chemokines can theoretically affect the tumorigenesis, progression, metastasis formation, or patients’ outcomes, the expression and prognostic value of CXC chemokines in UCEC have not been previously investigated.

The present study represented a comprehensive analysis of the expression profile, prognostic value of CXC chemokines in UCEC, and evaluated their correlation with the abundance of immune infiltration, as well as the expression of immune checkpoint inhibitors (ICIs). Our findings provided an overview of the potential role of CXC chemokines in the prognosis evaluation, and the possibility of applying CXCL2/12/14/17 expression levels for the selection of patients for perspective immunotherapeutic interventions.

## Materials and methods

### TCGA dataset analysis

The Cancer Genome Atlas (TCGA, https://portal.gdc.cancer.gov/) was used to analyze the expression profile of CXC chemokines in UCEC. A total of 425 files, containing tumor samples and adjacent non-tumor samples, were selected from the TCGA-GDC database. Gene expression data was visualized by using R (version 4.0.3) package of “limma”, and the statistical significance was obtained by Wilcox tests.

### UALCAN database analysis

UALCAN database (http://ualcan.path.uab.edu/analysis.html) contains publicly available high-throughput mRNA sequencing information (RNA-seq), clinical details, and survival data on 31 forms of cancer. The expression levels of CXCL2/12/14/17 in UCEC samples were analyzed by entering gene symbol(s) “CXCL2/12/14/17” with TCGA dataset “Uterine corpus endometrial carcinoma”, and selecting the “Expression” in the “Links for analysis” module. Correlations between CXCL2/12/14/17 expression and the clinicopathological parameters of UCEC patients were conducted based on the information contained in the “individual cancer stages”, “patients’ age”, “patients’ weight” and “menopause status” fields.

### Kaplan-Meier Plotter database analysis

Kaplan-Meier Plotter database (http://kmplot.com/analysis/index.php?p=background) is an online tool that can be utilized to analyze the impact of more than 54000 genes on the clinical outcomes in diverse cancers. For the purpose of survival analysis, patients with UCEC from the TCGA dataset were classified into high and low risk groups based on the optimal cutoff value of CXCL2/12/14/17 expression, and the overall survival and disease free survival were evaluated with logrank P-value and Hazard Ratio (HR) with 95% Confidence Interval (CI) by entering “Gene symbol”, “Survival” and “Uterine corpus endometrial carcinoma”. To evaluate the effect on the abundance of immune cell subgroups, the database search was limited by selecting the “Restrict analysis based on cellular content” option before the generation of Kaplan-Meier (KM) survival curves.

### cBioPortal database analysis

The cBio Cancer Genomics Portal (cBioPortal, http://www.cbioportal.org/) is an open-access web-based resource for the muti-dimensional analysis of cancer genomics data. It contains information on more than 5000 tumor samples representing 20 kinds of cancer. The genetic alteration frequencies of CXCL2/12/14/17 in UCEC (TCGA, PanCancer Atlas, 529 samples) were acquired by using the “OncoPrint” module. The influence of genetic alterations of CXCL2/12/14/17 on the overall survival and disease free survival of UCEC patients was analyzed with the “Comparison/Survival” module.

### TIMER2.0 database analysis

Tumor Immune Estimation Resource 2.0 (TIMER2.0, http://timer.cistrome.org/) provides comprehensive resources for the systematic analysis of immune infiltration for diverse cancer types from TCGA. It includes 10897 samples derived from 32 forms of cancer. Associations between CXCL2/12/14/17 expression and the abundance of infiltrating immune cells, including B cells, CD8+ T cells, CD4+ T cells, macrophages, neutrophils, and dendritic cells, were carried out with the “Gene” module.

### TISIDB database analysis

TISIDB database (http://cis.hku.hk/TISIDB/index.php) is an integrated web-based tool to analyze the interactions between tumors and the immune system. Correlations between CXCL2/12/14/17 expression and the abundance of tumor-infiltrating lymphocytes (TILs) in UCEC was analyzed by entering CXCL2/12/14/17 into the “Gene symbol” module and selecting the “Lymphocyte” module. Relationships between CXCL2/12/14/17 expression and the immunoinhibitors were carried out by entering CXCL2/12/14/17 into the “Gene symbol” module and selecting the “Immunomodulator” module.

### MethSurv database analysis

MethSurv database (https://biit.cs.ut.ee/methsurv/) is a useful tool for the visualization of DNA methylation. It was applied to determine DNA methylation sites within the genes coding for CXCL2/12/14/17 in UCEC samples, as well as the DNA methylation-based survival curves by using TCGA dataset. Heatmaps of DNA methylation of CXCL2/12/14/17 were created using the “Gene visualization” module, and differences in survival curves based on the distribution of methylation sites within CXCL2/12/14/17 genes in UCEC samples were acquired by using the “Single CpG” module.

## Results

### Differentially expressed CXC chemokines in UCEC

**Fig 1:**
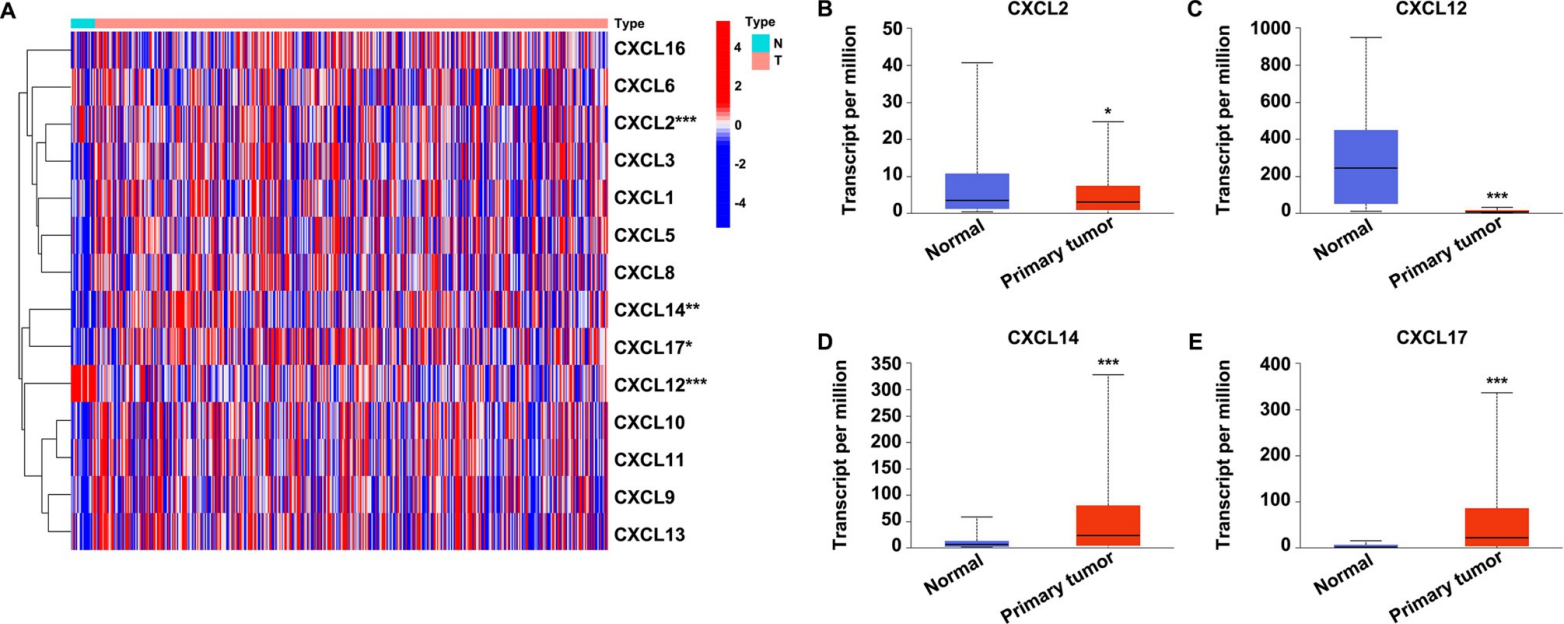
Expression profile of CXC chemokines in UCEC. A. The expression profile of CXC chemokines (CXCL1-17) in UCEC analyzed with TCGA dataset. B-E. The expression of CXCL2 (B), CXCL12 (C), CXCL14 (D), and CXCL17 (E) in UCEC were analyzed with the UALCAN database. *P<0.05, ***P<0.001.

The initial analysis of CXC chemokines (CXCL1-17) expression was carried out with a dataset of 425 UCEC samples downloaded from TCGA database. We found four members, CXCL2, CXCL12, CXCL14 and CXCL17, were differentially expressed (Fig 1A). The decreased expression of CXCL2 (Fig 1B), CXCL12 (Fig 1C), and increased expression of CXCL14 (Fig 1D), CXCL17 (Fig 1E) was further validated in UALCAN database.

### Relationships between CXCL2/12/14/17 expression and clinicopathological features of UCEC patients

**Fig 2:**
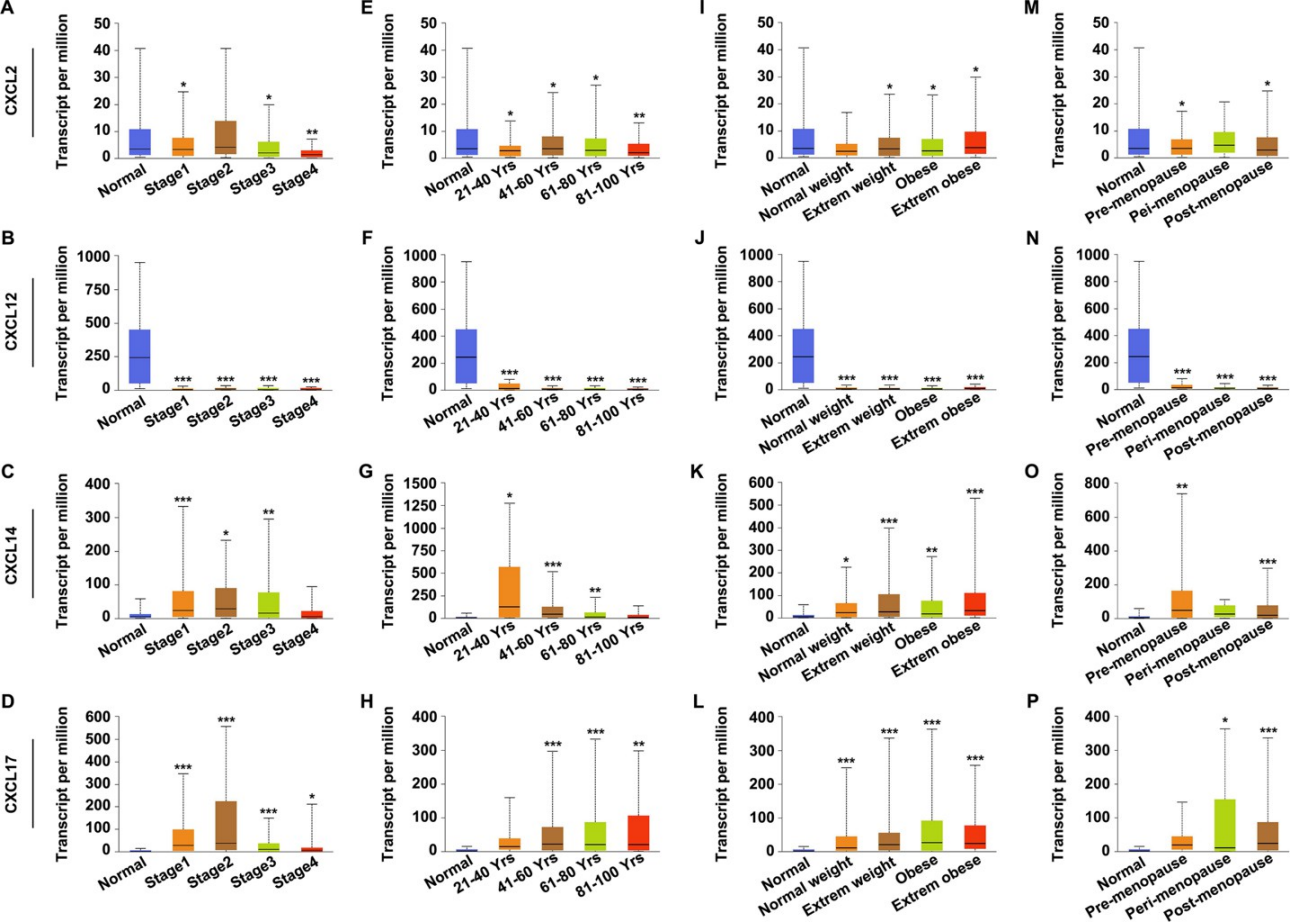
Correlations between the expression of CXCL2/12/14/17 and the clinicopathological parameters of UCEC. A-D. Relationships between CXCL2 (A), CXCL12 (B), CXCL14 (C), and CXCL17 (D) expression and cancer stage of UCEC. E-H. Correlations between CXCL2 (E), CXCL12 (F), CXCL14 (G), and CXCL17 (H) expression and patients’ age of UCEC. I-L. Relationships between CXCL2 (I), CXCL12 (J), CXCL14 (K), and CXCL17 (L) expression and patients’ weight of UCEC. M-P. Correlations between CXCL2 (M), CXCL12 (N), CXCL14 (O), and CXCL17 (P) expression and patients’ menopause status of UCEC. *P<0.05, **P<0.01, ***P<0.001.

Having observed the differential expression of CXCL2/12/14/17 in UCEC, we next analyzed whether changes in their expression patterns correlated with clinicopathological features of UCEC patients by using UALCAN database. A deduced expression of CXCL2/12 showed negative correlation with cancer stage (Fig 2A and 2B), patients’ age (Fig 2E and 2F), weight (Fig 2I and 2J) and menopause status (Fig 2M and 2N). In contrast, the increased expression of CXCL14/17 indicated positive correlation with cancer stage (Fig 2C and 2D), patients’ age (Fig 2G and 2H), weight (Fig 2K and 2L) and menopause status (Fig 2O and 2P). These findings suggested that the differential expression of CXCL2/12/14/17 might be an underlying indicator which reflected the clinical characteristics of UCEC.

### The prognostic value and the impacts of the genetic alterations of CXCL2/12/14/17 on the outcomes of UCEC

**Fig 3:**
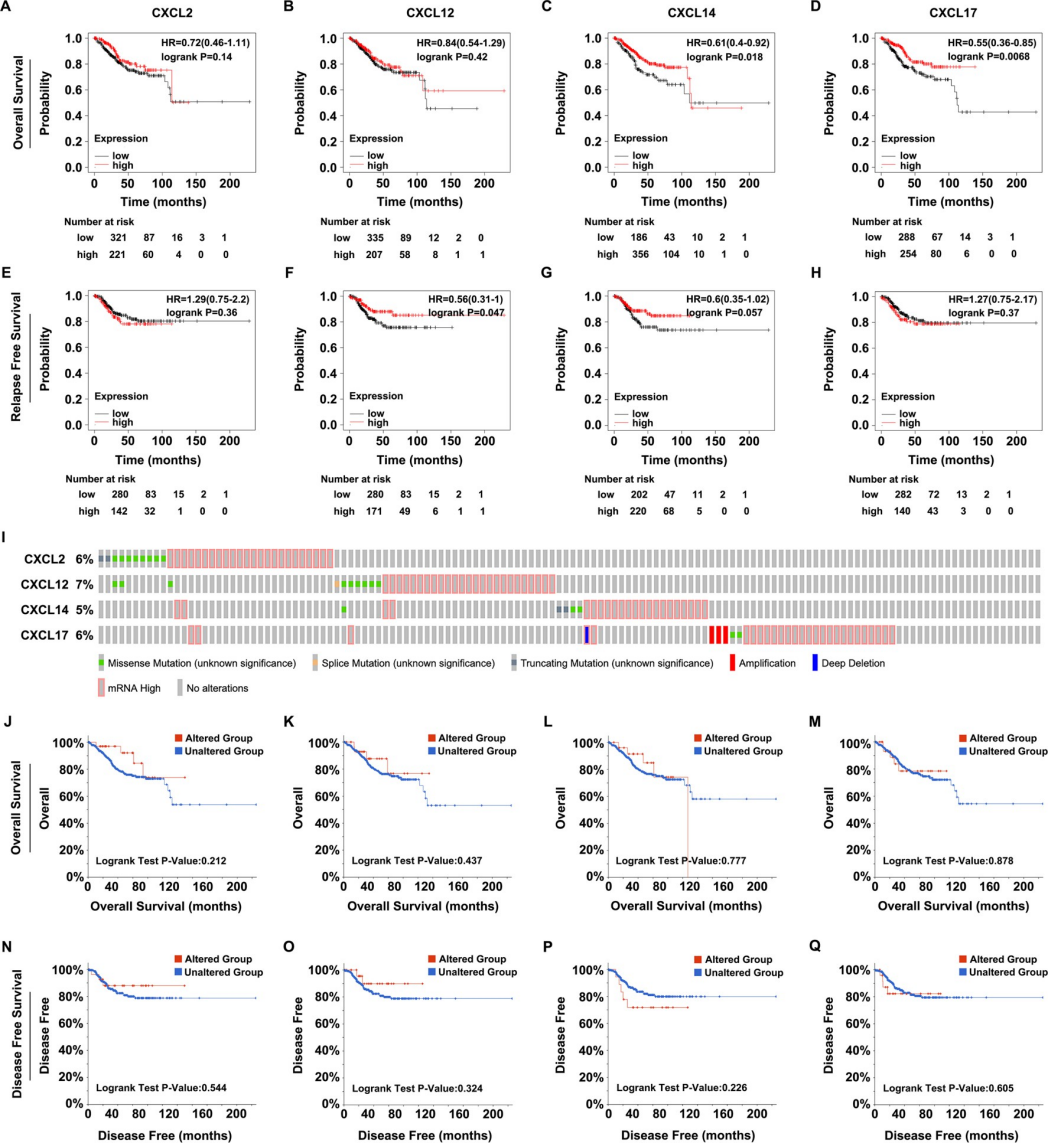
The prognostic value of CXCL2/12/14/17 for UCEC. A-D. The overall survival curves of CXCL2 (A), CXCL12 (B), CXCL14 (C), and CXCL17 (D) for UCEC analyzed in the Kaplan-Meier Plotter database. E-H. The disease free survival curves of CXCL2 (E), CXCL12 (F), CXCL14 (G), and CXCL17 (H) for UCEC analyzed in the Kaplan-Meier Plotter database. I. The genetic alteration frequencies of CXCL2/12/14/17 in UCEC analyzed in the cBioPortal database. J-M. The impacts of genetic alterations of CXCL2 (J), CXCL12 (K), CXCL14 (L), and CXCL17 (M) on the overall survival of UCEC analyzed in the cBioPortal database. N-Q. The effects of genetic alterations of CXCL2 (N), CXCL12 (O), CXCL14 (P), and CXCL17 (Q) on the disease free survival of UCEC analyzed in the cBioPortal database.

To investigate if there was a correlation between CXCL2/12/14/17 expression and clinical outcomes in UCEC patients, the Kaplan-Meier Plotter database was selected for the analysis. Higher CXCL14/17 expression levels were associated with better overall survival (Fig 3C and 3D), while CXCL2/12 expression levels represented no measurable influence on this outcome of UCEC patients (Fig 3A and 3B). In contrast, when patients were analyzed for the length of relapse free survival, individuals with higher CXCL12 expression showed better outcome than those with lower CXCL12 abundance (Fig 3F), whereas the expression of CXCL2/14/17 indicated no impact on the relapse free survival in UCEC patients (Fig 3E, 3G and 3H). In addition, we also explored the genetic alteration frequencies of CXCL2/12/14/17 in UCEC via the cBioPortal database. CXCL2 was altered in 34 out of 529 samples (6%), while CXCL12 was altered in 35 out of 529 samples (7%). CXCL14 was altered in 27 out of 529 samples (5%) and CXCL17 was altered in 32 out of 529 (6%) of UCEC patients. The detected alterations included missense, splice-site and truncating mutations, amplification, deep deletion, and mRNA high (Fig 3I). However, the genetic alterations of CXCL2/12/14/17 had no significant effect on the overall survival (Fig 3J–3M) and disease free survival (Fig 3N–3Q) in UCEC patients. Altogether, these findings elucidated that CXCL2/12 expression decreased, while CXCL14/17 expression increased in UCEC. Furthermore, the detected alterations in CXCL12/14/17 expression represented significant correlation with the clinical outcomes of UCEC patients.

### Associations between the expression of CXCL2/12/14/17 and the abundance of immune infiltration

**Fig 4:**
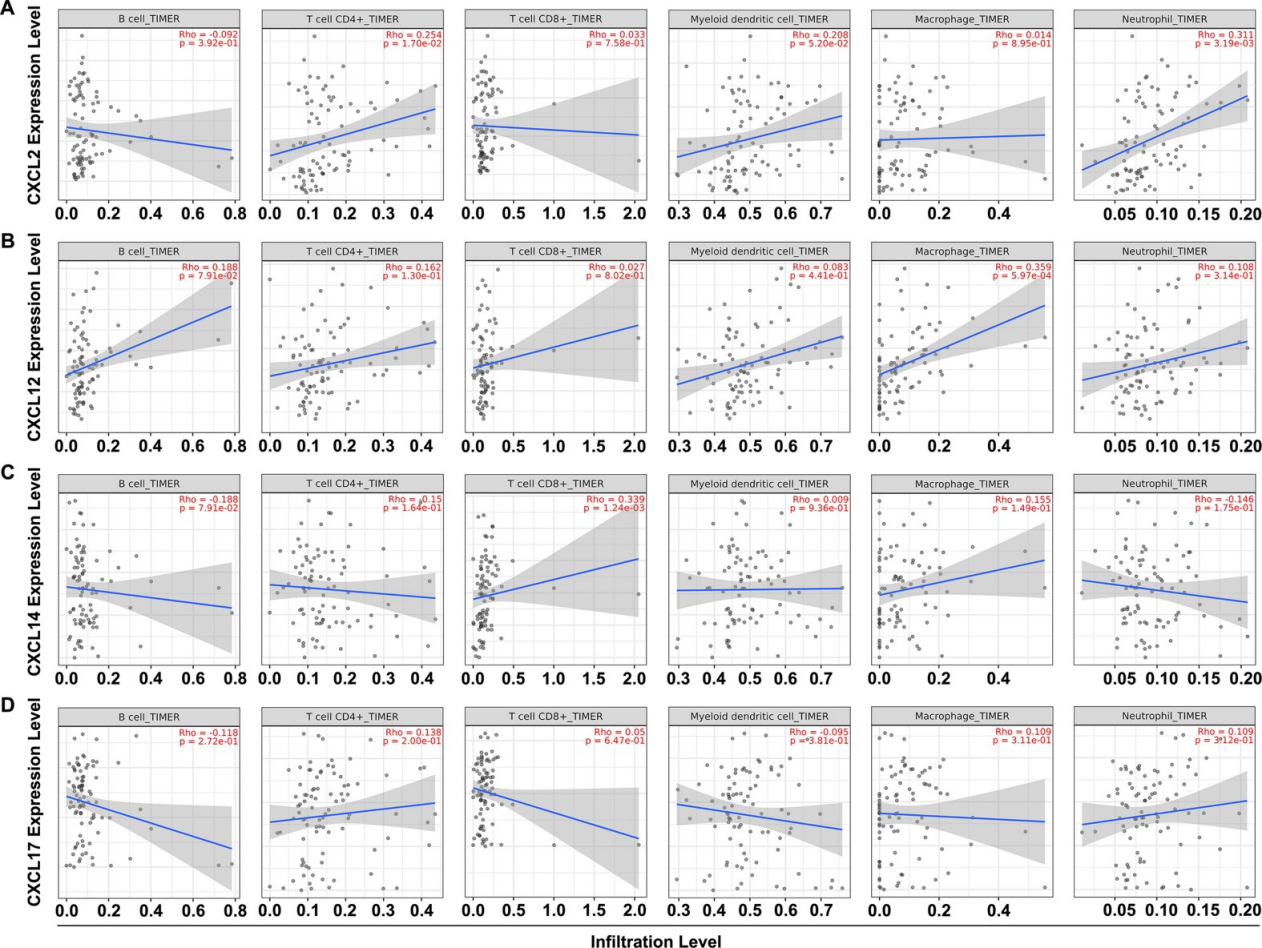
Associations between the expression of CXCL2/12/14/17 and the immune infiltration levels in UCEC. A-D. Associations between the expression of CXCL2 (A), CXCL12 (B), CXCL14 (C), and CXCL17 (D) and the immune infiltration levels in UCEC analyzed in the TIMER2.0 database.

**Fig 5:**
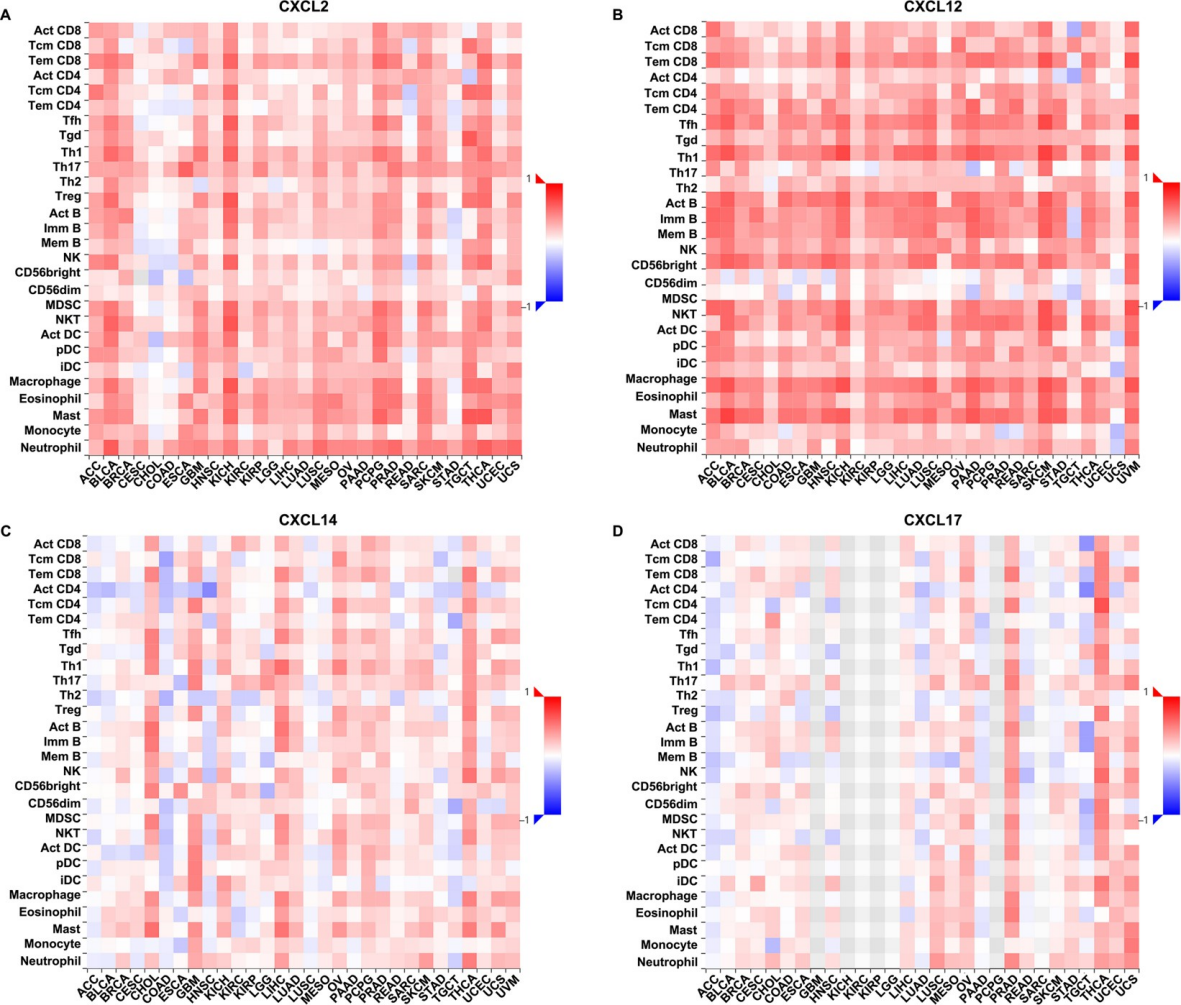
Relationships between the expression of CXCL2/12/14/17 and the abundance of tumor-infiltrating lymphocytes in UCEC. A-D. Correlations between CXCL2 (A), CXCL12 (B), CXCL14 (C), and CXCL17 (D) expression and the abundance of 28 kinds of tumor-infiltrating lymphocytes analyzed in the TISIDB database.

Given the essential role of CXC chemokines in the induction, activation and recruitment of immune cells, we used TIMER2.0 database to explore how changes in CXCL2/12/14/17 expression altered the extent of immune infiltration levels in UCEC. The results revealed a positive correlation between CXCL2 expression and CD4+ T cells (Rho = 0.254, p = 1.70e-02) and neutrophils (Rho = 0.311, p = 3.19e-03) (Fig 4A). At the same time, increased CXCL12 expression was only associated with macrophages (Rho = 0.359, p = 5.97e-04) (Fig 4B), and CXCL14 level showed positive correlation with CD8+ T cells (Rho = 0.339, p = 1.24e-03) (Fig 4C). However, no significant correlation was observed between CXCL17 expression and the abundance of immune infiltration (Fig 4D). In addition, we applied the TISIDB database to establish a detailed relationship between the expression of CXCL2/12/14/17 and the abundance of 28 distinct tumor-infiltrating lymphocytes subsets (Fig 5A–5D). As shown in Table 1, changes in CXCL2 expression was positively correlated with 22 kinds (Act CD8, Tem CD8, Tcm CD4, Tfh, Tgd, Th1, Th17, Th2, Act B, Imm B, NK, CD56bright, MDSC, NKT, Act DC, pDC, iDC, Macrophage, Eosinophil, Mast, Monocyte, Neutrophil), while the presence of 21 subsets of tumor-infiltrating lymphocytes indicated a positive correlation with CXCL12 expression (Act CD8, Tcm CD8, Tem CD8, Tcm CD4, Tem CD4, Tfh, Tgd, Th1, Th2, Treg, Act B, Imm B, Mem B, NK, MDSC, NKT, pDC, Macrophage, Eosinophil, Mast, Neutrophil). CXCL14 expression influenced the recruitment of 15 subpopulations (Act CD8, Tcm CD4, Tgd, Th17, Th2, NK, CD56bright, MDSC, NKT, pDC, iDC, Macrophage, Eosinophil, Mast, Neutrohpil), whereas CXCL17 expression correlated positively with the presence of 19 kinds of tumor-infiltrating lymphocytes (Act CD8, Tem CD8, Tcm CD4, Tfh, Th17, Act B, Imm B, NK, CD56bright, MDSC, NKT, Act DC, pDC, iDC, Macrophage, Eosinophil, Mast, Monocyte, Neutrophil). Furthermore, upregulated CXCL14/17 expression showed negative correlation with the abundance of three tumor-infiltrating lymphocytes (CXCL14: Tcm CD8, Act CD4, CD56dim; CXCL17: Tcm CD8, Act CD4, Tem CD4). These data provided strong evidence for the significant roles of CXCL2/12/14/17 in influencing immune responses within the tumor microenvironment in UCEC.

**Table 1:** Correlations between the expression of CXCL2/12/14/17 and the abundance of tumor-infiltrating lymphocytes.

	CXCL2		CXCL12		CXCL14		CXCL17	
Cell name	rho	*P*-value	rho	*P*-value	rho	*P*-value	rho	*P*-value
**Act CD8**	**0.101**	0.0179*	**0.208**	1.03e-06***	**0.11**	0.0104*	**0.1**	0.0193*
**Tcm CD8**	-0.02	0.644	**0.259**	9.39e-10***	**-0.118**	0.00564**	**-0.103**	0.0162*
**Tem CD8**	**0.282**	2.45e-11***	**0.342**	2.18e-16***	0.051	0.231	**0.172**	5.6e-05***
**Act CD4**	-0.003	0.935	0.043	0.315	**-0.164**	0.000116***	**-0.193**	5.49e-06***
**Tcm CD4**	**0.135**	0.00157**	**0.24**	1.49e-08***	**0.157**	0.000231***	**0.106**	0.0129*
**Tem CD4**	-0.062	0.151	**0.263**	5.38e-10***	-0.052	0.222	**-0.084**	0.0485*
**Tfh**	**0.157**	0.000245***	**0.388**	<2.2e-16***	0.076	0.076	**0.085**	0.0475*
**Tgd**	**0.092**	0.0316*	**0.104**	0.0152*	**0.177**	3.43e-05***	-0.055	0.199
**Th1**	**0.244**	8.6e-09***	**0.436**	<2.2e-16***	0.036	0.396	0.081	0.0585
**Th17**	**0.387**	<2.2e-16***	0.055	0.201	**0.208**	9.51e-07***	**0.282**	2.35e-11***
**Th2**	**0.11**	0.0104*	**0.166**	9.5e-05***	**0.096**	0.025*	-0.055	0.198
**Treg**	0.082	0.0544	**0.336**	8.66e-16***	0.036	0.408	-0.014	0.749
**Act B**	**0.187**	1.17e-05***	**0.407**	<2.2e-16***	-0.035	0.409	**0.181**	2.22e-05***
**Imm B**	**0.16**	0.00018***	**0.348**	5.13e-17***	-0.082	0.0557	**0.154**	0.000306***
**Mem B**	0.026	0.542	**0.176**	3.56e-05***	-0.059	0.171	-0.056	0.189
**NK**	**0.161**	0.000155***	**0.409**	<2.2e-16***	**0.118**	0.00589**	**0.089**	0.0368*
**CD56bright**	**0.187**	1.19e-05***	0.048	0.268	**0.247**	5.95e-09***	**0.209**	9.22e-07***
**CD56dim**	0.053	0.213	0.003	0.938	**-0.125**	0.00339**	0.046	0.285
**MDSC**	**0.215**	4.22e-07***	**0.266**	3.35e-10***	**0.107**	0.0126*	**0.171**	5.91e-05***
**NKT**	**0.166**	0.000104***	**0.438**	<2.2e-16***	**0.117**	0.00602**	**0.101**	0.0178*
**Act DC**	**0.236**	2.72e-08***	0.081	0.0595	-0.067	0.119	**0.16**	0.000174***
**pDC**	**0.198**	3.3e-06***	**0.244**	8.6e-09***	**0.087**	0.0422*	**0.265**	3.66e-10***
**iDC**	**0.283**	1.89e-11***	0.039	0.357	**0.114**	0.0079**	**0.249**	3.95e-09***
**Macrophage**	**0.29**	6e-12***	**0.378**	<2.2e-16***	**0.141**	0.001**	**0.197**	3.68e-06***
**Eosinophil**	**0.414**	<2.2e-16***	**0.299**	1.2e-12***	**0.201**	2.42e-06***	**0.291**	4.98e-12***
**Mast**	**0.265**	4e-10***	**0.401**	<2.2e-16***	**0.175**	4.15e-05***	**0.245**	6.91e-09***
**Monocyte**	**0.203**	1.73e-06***	0.073	0.0878	0.023	0.597	**0.17**	6.96e-05***
**Neutrophil**	**0.54**	<2.2e-16***	**0.1**	0.0191*	**0.116**	0.0065**	**0.323**	1.46e-14***

Notes: The expression of CXCL2 was positively correlated with 22 kinds of TILs and CXCL12 was 21 kinds, whereas CXCL14 was 15 kinds and CXCL17 was 19 kinds. Oppositely, the expression of CXCL14/17 was negatively correlated with 3 kinds of TILs.

*P<0.05,

**P<0.01,

***P<0.001

### Correlations between the abundance of CXCL2/12/14/17 and the expression of immune checkpoint inhibitors in UCEC

**Fig 6:**
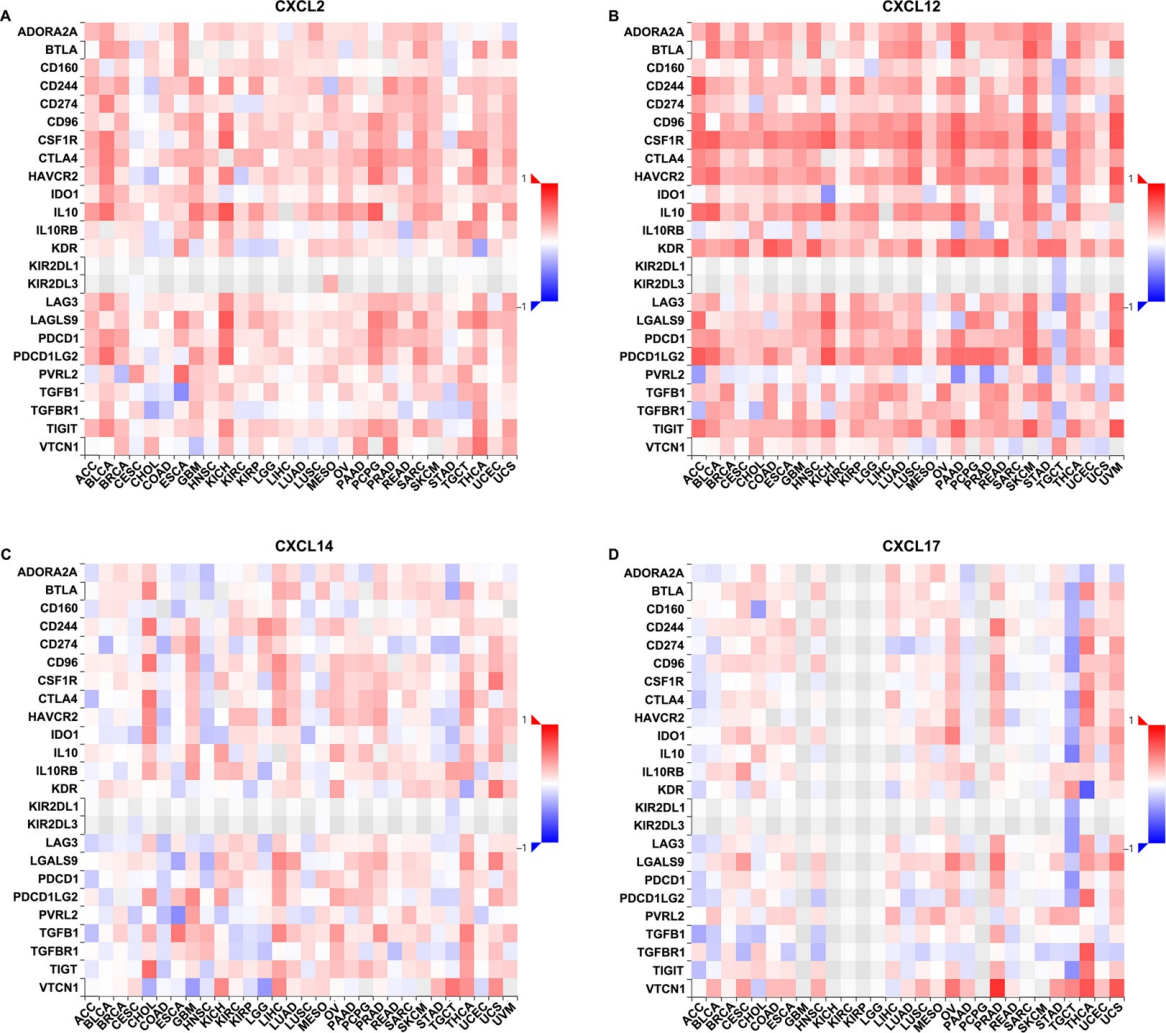
Correlations between the expression of CXCL2/12/14/17 and the abundance of immunoinhibitors in UCEC. A-D. Associations between CXCL2 (A), CXCL12 (B), CXCL14 (C), and CXCL17 (D) expression and the immunoinhibitors in UCEC analyzed in the TISIDB database.

Immune checkpoint inhibitors are naturally occurring proteins that control the extent of immune responses to specific stimuli. These proteins represent promising novel therapeutic targets in the management of certain forms of cancer. Therefore, we explored whether the detected alterations in CXC chemokines affected the expression of immune checkpoint inhibitors. The relationships between the expression of CXCL2/12/14/17 and the abundance of 24 immunoinhibitors were assessed in UCEC (Fig 6A–6D), and some particularly strong correlations were detected. As shown in Table 2, CXCL2 level was positively correlated with 11 immunoinhibitors which were BTLA (rho = 0.085, P-value = 0.0464), CD244 (rho = 0.199, P-value = 2.76e-06), CD274 (rho = 0.113, P-value = 0.00816), CD96 (rho = 0.196, P-value = 3.91e-06), CSF1R (rho = 0.19, P-value = 8.41e-06), CTLA4 (rho = 0.159, P-value = 0.000193), HAVCR2 (rho = 0.106, P-value = 0.0136), IDO1 (rho = 0.226, P-value = 1.02e-07), KDR (rho = 0.15, P-value = 0.000427), LGALS9 (rho = 0.274, P-value = 8.71e-11) and VTCN1 (rho = 0.141, P-value = 0.000945); CXCL12 was correlated with 17 immunoinhibitors which were ADORA2A (rho = 0.306, P-value = 3.73e-13), BTLA (rho = 0.262, P-value = 6.11e-10), CD160 (rho = 0.126, P-value = 0.00319), CD244 (rho = 0.254, P-value = 1.86e-09), CD96 (rho = 0.309, P-value = 1.91e-13), CSF1R (rho = 0.35, P-value = 3.61e-17), CTLA4 (rho = 0.234, P-value = 3.4e-08), HAVCR2 (rho = 0.25, P-value = 3.5e-09), IL10 (rho = 0.164, P-value = 0.000116), KDR (rho = 0.411, P-value<2.2e-16), LAG3 (rho = 0.196, P-value = 4.07e-06), PDCD1 (rho = 0.247, P-value = 5.84e-09), PDCD1LG2 (rho = 0.289, P-value = 7.69e-12), TGFB1 (rho = 0.329, P-value = 4.19e-15), TGFBR1 (rho = 0.087, P-value = 0.0427), TIGIT (rho = 0.256, P-value = 1.56e-09) and VTCN1 (rho = -0.119, P-value = 0.00541). Whereas CXCL14 was correlated with 9 immunoinhibitors which were CD244 (rho = 0.127, P-value = 0.00308), CD96 (rho = 0.12, P-value = 0.00513), CTLA4 (rho = 0.218, P-value = 2.96e-07), IL10 (rho = 0.12, P-value = 0.00516), IL10RB (rho = -0.179, P-value = 2.78e-05), KDR (rho = 0.095, P-value = 0.0272), PDCD1 (rho = 0.126, P-value = 0.00314), PVRL2 (rho = -0.19, P-value = 8.3e-06) and VTCN1 (rho = -0.15, P-value = 0.000447); CXCL17 was correlated with 14 immunoinhibitors which were BTLA (rho = 0.101, P-value = 0.0182), CD160 (rho = 0.085, P-value = 0.0478), CD244 (rho = 0.177, P-value = 3.38e-05), CD96 (rho = 0.217, P-value = 3.33e-07), CSF1R (rho = 0.156, P-value = 0.000258), HAVCR2 (rho = 0.086, P-value = 0.0449), IDO1 (rho = 0.148, P-value = 0.000526), IL10 (rho = -0.127, P-value = 0.00287), KDR (rho = 0.087, P-value = 0.043), LAG3 (rho = -0.109, P-value = 0.0109), LGALS9 (rho = 0.27, P-value = 1.79e-10), TGFB1 (rho = -0.134, P-value = 0.00176), TGFBR1 (rho = -0.159, P-value = 0.000195) and VTCN1 (rho = 0.235, P-value = 3.22e-08). These data illustrated that CXCL2/12/14/17 was likely to be involved in tumor-specific immune responses by regulating the infiltration of immune cells and by modulating the expression of crucial molecules with essential immunoregulatory functions.

**Table 2:** Associations between the expression of CXCL2/12/14/17 and the immune checkpoint inhibitors.

	CXCL2		CXCL12		CXCL14		CXCL17	
Cell name	Rho	*P*-value	rho	*P*-value	rho	*P*-value	rho	*P*-value
**ADORA2A**	0.039	0.362	**0.306**	3.73e-13***	-0.022	0.609	0.031	0.474
**BTLA**	**0.085**	0.0464*	**0.262**	6.11e-10***	0.044	0.302	**0.101**	0.0182*
**CD160**	0.053	0.215	**0.126**	0.00319**	-0.015	0.732	**0.085**	0.0478*
**CD244**	**0.199**	2.76e-06***	**0.254**	1.86e-09***	**0.127**	0.00308**	**0.177**	3.38e-05***
**CD274**	**0.113**	0.00816**	0.033	0.445	-0.029	0.503	0.029	0.493
**CD96**	**0.196**	3.91e-06***	**0.309**	1.91e-13***	**0.12**	0.00513**	**0.217**	3.33e-07***
**CSF1R**	**0.19**	8.41e-06***	**0.35**	3.61e-17***	0.056	0.191	**0.156**	0.000258***
**CTLA4**	**0.159**	0.000193***	**0.234**	3.4e-08***	**0.218**	2.96e-07***	0.082	0.0548
**HAVCR2**	**0.106**	0.0136*	**0.25**	3.5e-09***	0.018	0.669	**0.086**	0.0449*
**IDO1**	**0.226**	1.02e-07***	0.034	0.425	-0.059	0.168	**0.148**	0.000526***
**IL10**	0.03	0.479	**0.164**	0.000116***	**0.12**	0.00516**	**-0.127**	0.00287**
**IL10RB**	0.001	0.978	0.034	0.431	**-0.179**	2.78e-05***	0.074	0.0828
**KDR**	**0.15**	0.000427***	**0.411**	<2.2e-16***	**0.095**	0.0272*	**0.087**	0.043*
**LAG3**	-0.066	0.121	**0.196**	4.07e-06***	-0.029	0.506	**-0.109**	0.0109*
**LGALS9**	**0.274**	8.71e-11***	0.066	0.123	0.05	0.243	**0.27**	1.79e-10***
**PDCD1**	0.045	0.297	**0.247**	5.84e-09***	**0.126**	0.00314**	-0.026	0.549
**PDCD1LG2**	-0.016	0.707	**0.289**	7.69e-12***	-0.003	0.939	-0.052	0.227
**PVRL2**	-0.002	0.967	-0.007	0.877	**-0.19**	8.3e-06***	-0.047	0.274
**TGFB1**	0.032	0.455	**0.329**	4.19e-15***	0.058	0.176	**-0.134**	0.00176**
**TGFBR1**	-0.031	0.465	**0.087**	0.0427*	0.052	0.223	**-0.159**	0.000195***
**TIGIT**	0.045	0.296	**0.256**	1.56e-09***	-0.006	0.888	0.024	0.573
**VTCN1**	**0.141**	0.000945***	**-0.119**	0.00541**	**-0.15**	0.000447***	**0.235**	3.22e-08***

Notes: The expression of CXCL2 was positively correlated with 11 kinds of ICIs, and CXCL12 was 17 kinds, whereas CXCL14 was positively associated with 9 kinds of ICIs and CXCL17 was 14 kinds.

*P<0.05,

**P<0.01,

***P<0.001

### DNA methylation heatmaps of CXCL2/12/14/17 and the prognostic value in the clinical management of UCEC

**Fig 7:**
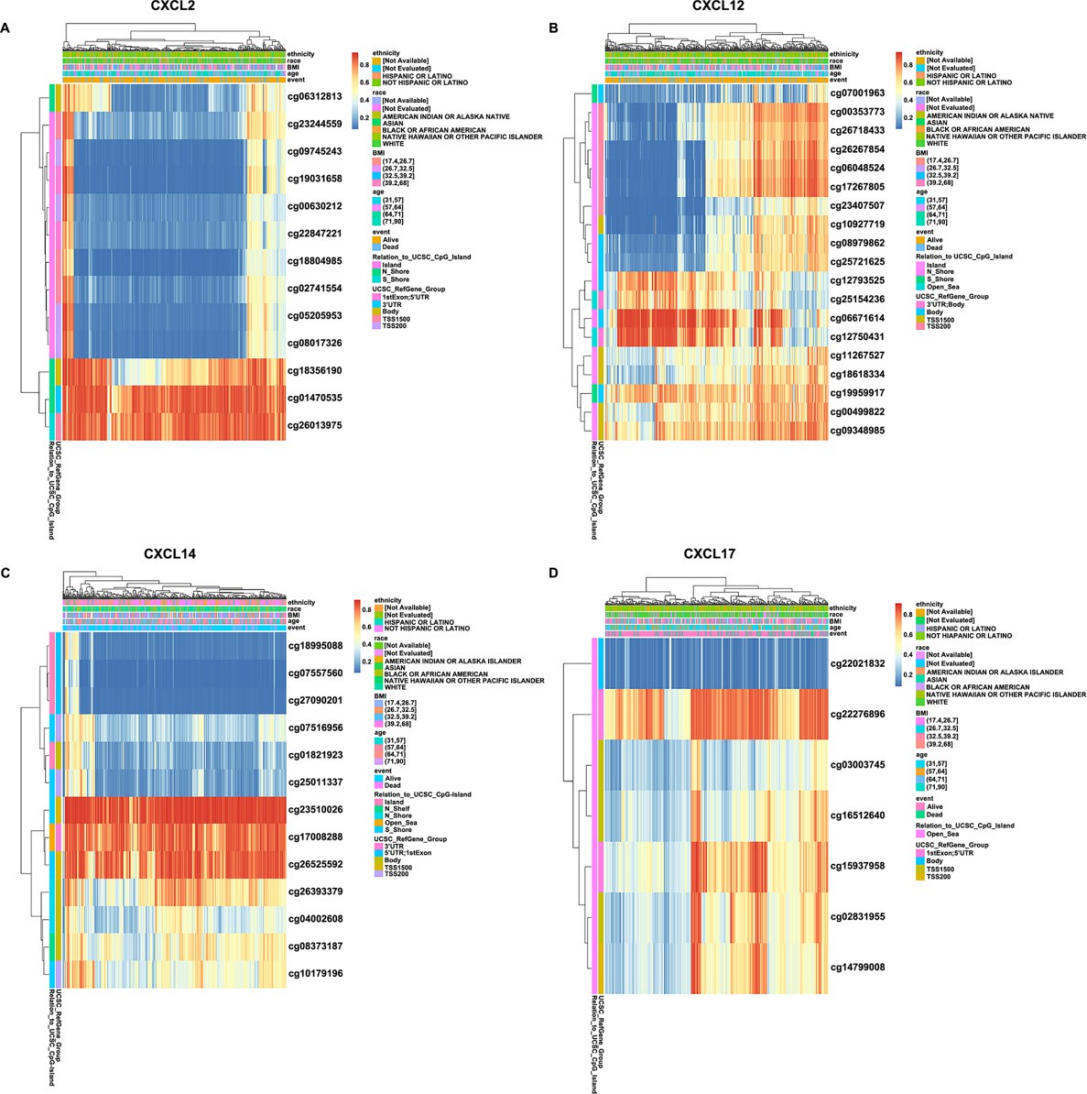
DNA methylation heatmaps of CXCL2/12/14/17 in UCEC. A-D. Heatmaps of DNA methylation of CXCL2 (A), CXCL12 (B), CXCL14 (C), and CXCL17 (D) in UCEC analyzed in the MethSurv database. Red: high expression; Blue: low expression.

DNA methylation plays a pivotal role in altering gene expression patterns and has been shown to influence clinical outcomes of cancer patients. Therefore, we explored if changes in CXCL2/12/14/17 expression were associated with the altered methylation of the corresponding chromosomal regions. We also investigated whether any of the detected alterations in methylation patterns correlated with the clinical behavior of the cancers, potentially providing prognostic information in UCEC by using the MethSurv database. The analysis identified 13 CpG methylation sites in the vicinity of the CXCL2 gene (Fig 7A), 19 CpG methylation sites in CXCL12 (Fig 7B), 13 CpG methylation sites in CXCL14 (Fig 7C), and 7 CpG methylation sites in CXCL17 (Fig 7D). Amongst these, cg01470535 and cg26013975 of CXCL2, cg06671614 and cg12750431 of CXCL12, cg23510026, cg17008288 and cg26525592 of CXCL14, cg22276896 of CXCL17 showed unexpectedly high levels of DNA methylation. When methylation levels were analyzed against clinical outcomes, we found 4 CpG sites around CXCL2, 16 CpG sites around CXCL12, and 3 CpG sites around CXCL14/17 where altered methylation appeared to affect tumor behavior and patients’ outcomes (Table 3). Methylation levels at 3 CpG sites of CXCL2 (cg01470535, cg05205953 and cg09745243), 4 CpG sites of CXCL12 (cg06671614, cg12793525, cg12750431 and cg25154236), 3 CpG sites of CXCL14 (cg18995088, cg04002608 and cg17008288) and CXCL17 (cg02831955, cg03003745 and cg16512640) corresponded to poorer outcomes, while there was a single CpG site of CXCL2 (cg18804985) and 12 CpG sites within CXCL12 (cg00353773, cg06048524, cg17267805, cg23407507, cg26267854, cg26718433, cg09348985, cg10927719, cg18618334, cg08979862, cg25721625 and cg07001963) where altered methylation improved outcomes. Altogether, these results demonstrated that DNA methylation of the genetic areas encoding CXCL2/12/14/17 played critical roles in the development and progression of UCEC, which showed a close correlation with the patients’ prognosis.

**Table 3:** Survival curves based on the methylation of CXCL2/12/14/17 in UCEC by the MethSurv database.

Name	CpG site	HR	*P*-value
**CXCL2**	3’UTR-N_Shore-cg01470535	1.896	0.01*
	TSS200-Island-cg05205953	1.777	0.038*
	TSS200-Island-cg09745243	2.66	0.0013**
	TSS1500-Island-cg18804985	0.618	0.042*
**CXCL12**	TSS200-Island-cg00353773	0.425	3e-04***
	TSS200-Island-cg06048524	0.464	0.0011**
	TSS200-Island-cg17267805	0.461	0.001**
	TSS200-Island-cg23407507	0.45	0.00073***
	TSS200-Island-cg26267854	0.443	0.00058***
	TSS200-Island-cg26718433	0.513	0.0085**
	TSS1500-Island-cg09348985	0.605	0.032*
	TSS1500-Island-cg10927719	0.439	0.00049***
	TSS1500-Island-cg18618334	0.539	0.0098**
	Body-Island-cg08979862	0.482	0.0023**
	Body-Island-cg25721625	0.484	0.002**
	Body-N_Shelf-cg06671614	1.852	0.0099**
	Body-N_Shelf-cg12793525	1.834	0.015*
	Body-N_Shore-cg07001963	0.499	0.011*
	3’UTR;Body-Open_Sea-cg12750431	2.156	0.0011**
	3’UTR;Body-Open_Sea-cg25154236	2.814	1.3e-05***
**CXCL14**	5’UTR;1stExon-Island-cg18995088	2.544	0.0033**
	Body-N_Shore-cg04002608	2.005	0.0034**
	3’UTR-Open_Sea-cg17008288	3.454	0.00013***
**CXCL17**	TSS1500-Open_Sea-cg02831955	1.666	0.03*
	TSS1500-Open_Sea-cg03003745	1.906	0.0063**
	TSS1500-Open_Sea-cg16512640	2.147	0.0012**

Notes: CXCL2 had 4 CpGs showed impacts on the outcomes of UCEC patients and CXCL12 had 16, whereas CXCL14/17 both had 3 CpGs represented influence on the prognosis of UCEC patients.

*P<0.05,

**P<0.01,

***P<0.001

## Discussion

Chemokines are classified into four subfamilies, namely C, CXC, CC and CX3C. Of these, CXC chemokines as crucial and indispensable components producing and secreting by immune, stromal or tumor cells, are mainly concentrated in the tumor microenvironment and have been implicated in the tumor-specific immune responses [23–25]. They can modulate the tumor microenvironment and biological phenotypes of the malignant cells, thus affecting clinical characteristics, patients’ outcomes and effectiveness of therapeutic interventions, including endometrial carcinoma [26], breast cancer [27], bladder cancer [28] and cervix cancer [29], etc.

UCEC is one of the commonest malignancies of the female reproductive system. It is also the leading cause of death amongst gynecological cancers due to its high recurrence rate and molecular heterogeneity [30–32]. Definitive diagnosis of UCEC currently relies on histological examination. Successful diagnose and management can save patients from consequent morbidity and premature mortality [33]. Advances in detection mean that most patients with UCEC can be diagnosed or treated at an early stage, improving survival. However, a significant number of patients are diagnosed at advanced stage or harbor occult metastatic lesions, causing tumor recurrence because of the limited response to therapies [34]. In addition, conventional diagnostic approaches provide limited prognostic information that could aid clinical judgement and provide therapeutic guidance [35]. Thus, to improve outcomes, it is urgent to identify novel, reliable, and accurate biomarkers for prognositic purposes and also as potential therapeutic targets for clinical interventions in UCEC. Here, we reported a computational approach for the detection of such potential biomarkers. Various cancer databases were searched and applied to investigate the expression profile and prognostic value of CXC chemokines in UCEC. We found that the expression of CXCL2/12 was significantly downregulated, while CXCL14/17 was upregulated in UCEC patients (Fig 1). Decreased expression of CXCL2/12 showed a negative, whereas increased expression of CXCL14/17 indicated a positive correlation with clinicopathological features of UCEC (Fig 2). Patients with higher CXCL14 expression experienced better overall survival while individuals with higher CXCL12 expression had a longer relapse free survival period (Fig 3). These findings suggested that CXC chemokines, especially CXCL12/14/17, might act as potential biomarkers for the prediction and evaluation of UCEC prognosis.

Cancer progression is a complex process that is influenced by extensive interactions between tumor cells, the microenvironment and the immune system [36]. There is accumulating evidence that the tumor microenvironment plays a critical role in supporting cancer progression and metastasis formation, with far reaching relevance in predicting clinical outcomes. In addition, immunological responses in the tumor microenvironment are potential targets in the immunotherapy of cancers [37–39]. As pivotal components of the tumor microenvironment, CXC chemokines act as a “ZIP code” for immune cells, guiding their influx into the tumor microenvironment to exert influences on cancer initiation and progression [40]. For example, it has been well documented that CXCL9/10/11 could effectively recruited infiltrating CD8+ T cells into the tumor microenvironment, which were regarded as potential cancer immune therapy targets [41–42]. The expression of SNAIL could induce epithelial-to-mesenchymal transition and lung metastasis of tumors secreting CXCL2 to promote the invasion of M2-type immunosuppressed macrophages in colorectal cancer. These findings elucidated the tumor-tumor associated macrophages interaction in the metastatic microenvironment mediated by tumor-derived CXCL2 [43]. CXCL12, derived from CD248-expressing cancer-associated fibroblasts, mediated M2-polarized macrophages to promote non-small cell lung cancer progression [44]. Exosomal CXCL14 contributed to M2 macrophage polarization through NF-kB signaling pathway to promote the progression of prostate cancer [45]. CXCL17-derived CD11b+Gr-1+ myeloid-derived suppressor cells contributed to lung metastasis of breast cancer through platelet-derived growth factor-BB [46]. Given the widespread role of CXC chemokines in oncogenesis, it was imperative to evaluate the possibility that CXCL2/12/14/17 could be differentially expressed in UCEC, potentially influencing the status of tumor microenvironment. For this purpose, we analyzed data from both the TIMER2.0 and TISIDB databases, and found that CXCL2 expression correlated with the influx of CD4+ T cells and neutrophils. CXCL12 was involved in the recruitment of macrophages, and CXCL14 caused the influx of CD8+ T cells (Fig 4). In a more detailed analysis, we found that among 28 subsets of tumor-infiltrating lymphocytes, 22 were correlated with CXCL2, 21 with CXCL12, 15 with CXCL14, and 19 with CXCL17 (Fig 5 and Table 1). In addition, the abundance of CXCL2/12/14/17 also influenced the expression of immune checkpoint inhibitors. Of the 24 kinds of immunoinhibitors present in the TISIDB database, CXCL2 was significantly correlated with 11 kinds and CXCL12 was 17 kinds of immunoinhibitors, whereas CXCL14 appeared to be significantly correlated with 9 kinds and CXCL17 was 14 kinds of immunoinhibitors in UCEC (Fig 6 and Table 2). The strong correlation between CXC chemokines and immune infiltration, as well as their impacts on the expression of immune checkpoint inhibitors, provided novel concepts and immunotherapeutic targets for UCEC patients.

In summary, we conducted a systematic analysis to evaluate the expression of CXC chemokines to determine their prognostic value and to analyze the correlations with immune infiltration, providing a comprehensive understanding of the molecular biological properties of UCEC. CXCL2/12 expression was decreased while CXCL14/17 was increased in UCEC. Higher expression of CXCL12/14/17 correlated with clinical patients’ outcomes. Moreover, a significant association between CXCL2/12/14/17 expression and clinicopathological parameters, as well as the immune infiltration levels in UCEC was observed. Thus, CXC chemokines were not only promising and valuable biomarkers for prognosis prediction and evaluation, but also potential immunotherapeutic targets due to the influence on immune status of UCEC.

## Supporting information

S1 File TCGA-UCEC dataset and R package for constructing heatmap of Fig 1A. (ZIP)

S2 File UALCAN database downloaded data for Figs 1 and 2. (ZIP)

S3 File Kaplan Meier Plotter database downloaded data for Fig 3A–3H. (ZIP)
