# Supplementary figures and images for: CXC chemokines: Potential biomarker and immunotherapeutic target for uterine corpus endometrial carcinoma
Source: PLoS One. 2024 Jan 17;19(1):e0277872. doi: 10.1371/journal.pone.0277872 (PMC10793908; doi:10.1371/journal.pone.0277872)

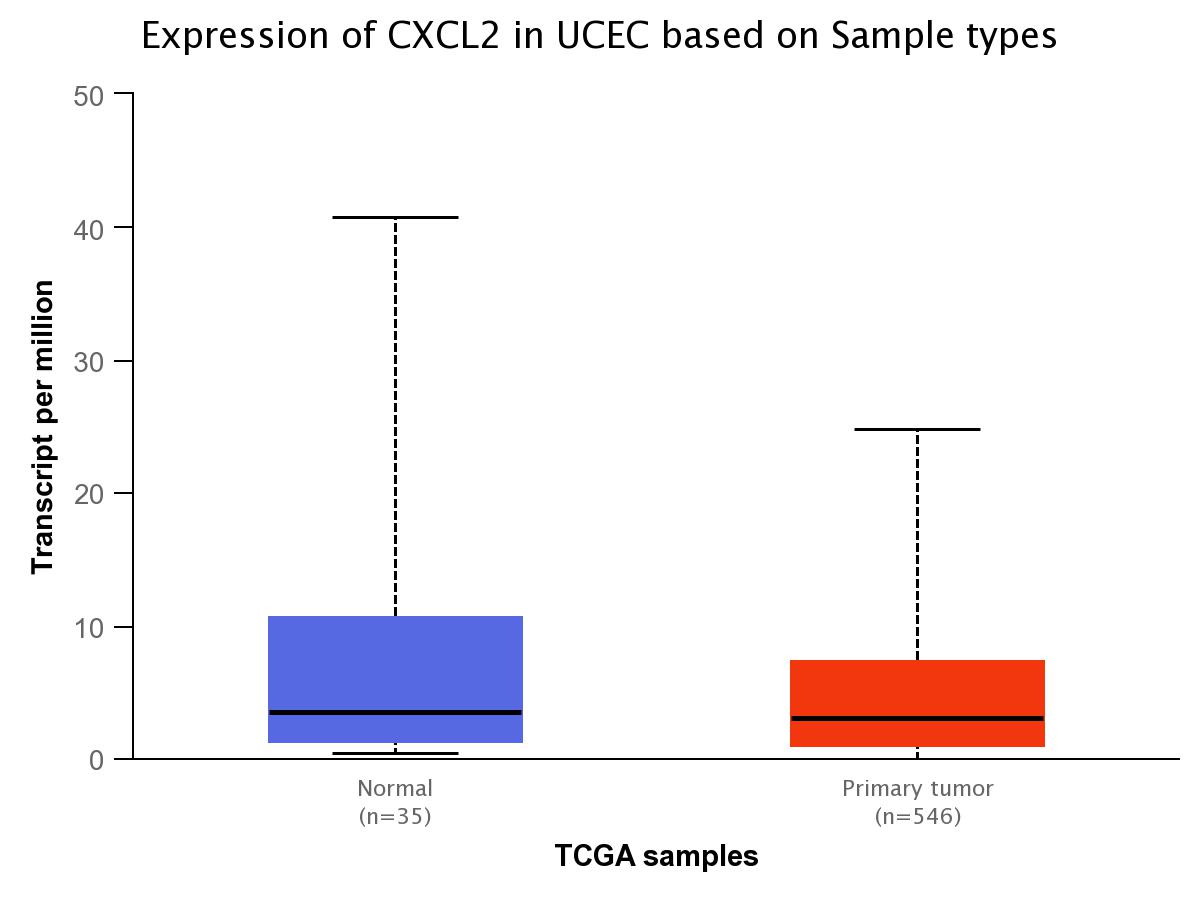

Supplement: S2 File — (ZIP) [file pone.0277872.s002.zip › S2_File/Figure 1B.jpeg]

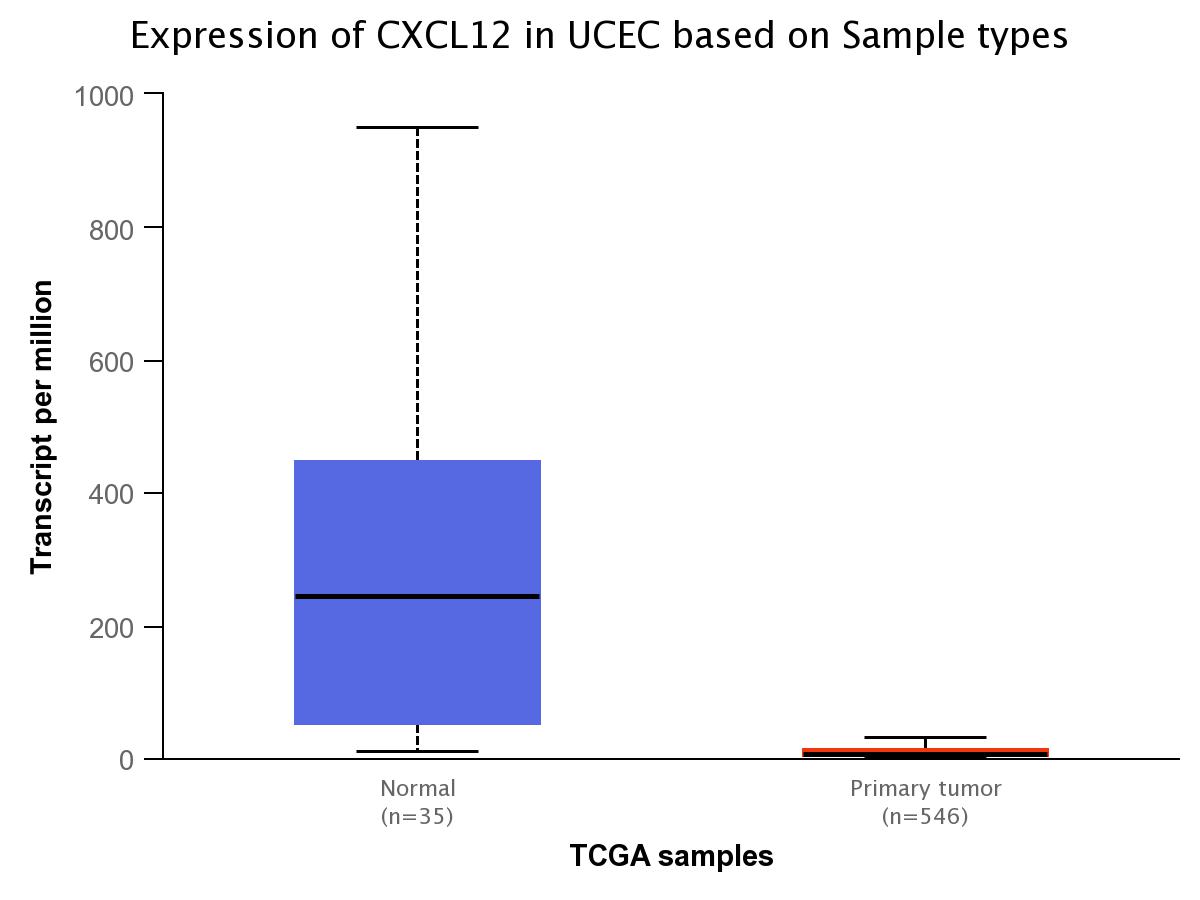

Supplement: S2 File — (ZIP) [file pone.0277872.s002.zip › S2_File/Figure 1C.jpeg]

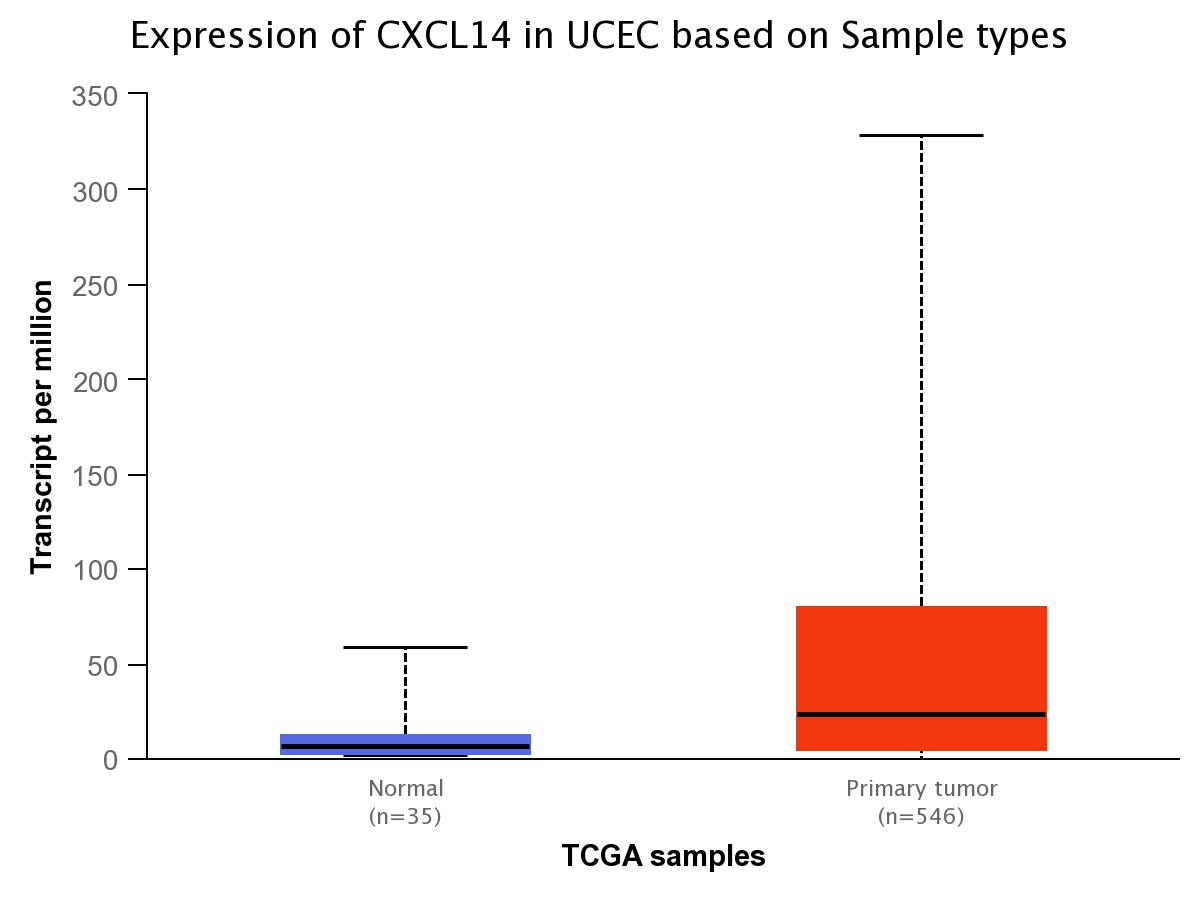

Supplement: S2 File — (ZIP) [file pone.0277872.s002.zip › S2_File/Figure 1D.jpeg]

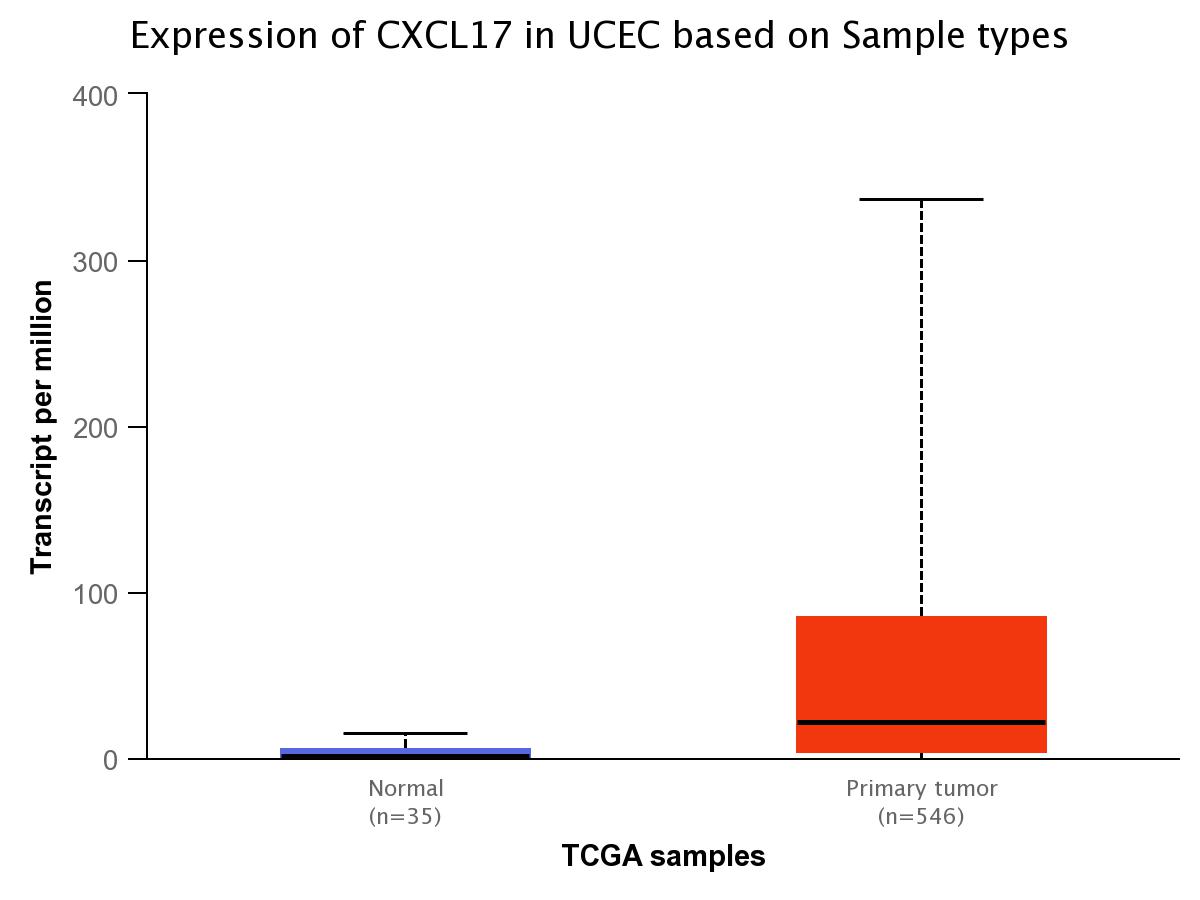

Supplement: S2 File — (ZIP) [file pone.0277872.s002.zip › S2_File/Figure 1E.jpeg]

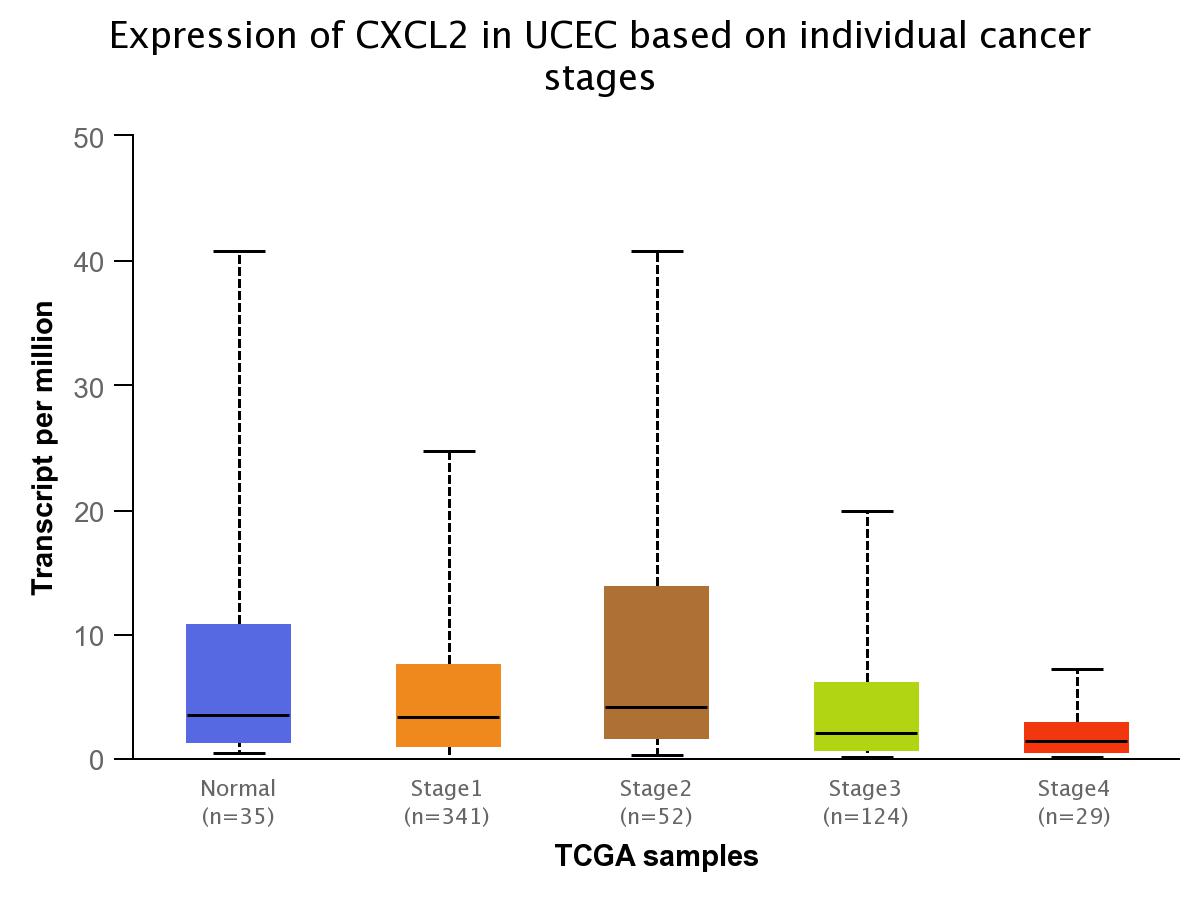

Supplement: S2 File — (ZIP) [file pone.0277872.s002.zip › S2_File/Figure 2A.jpeg]

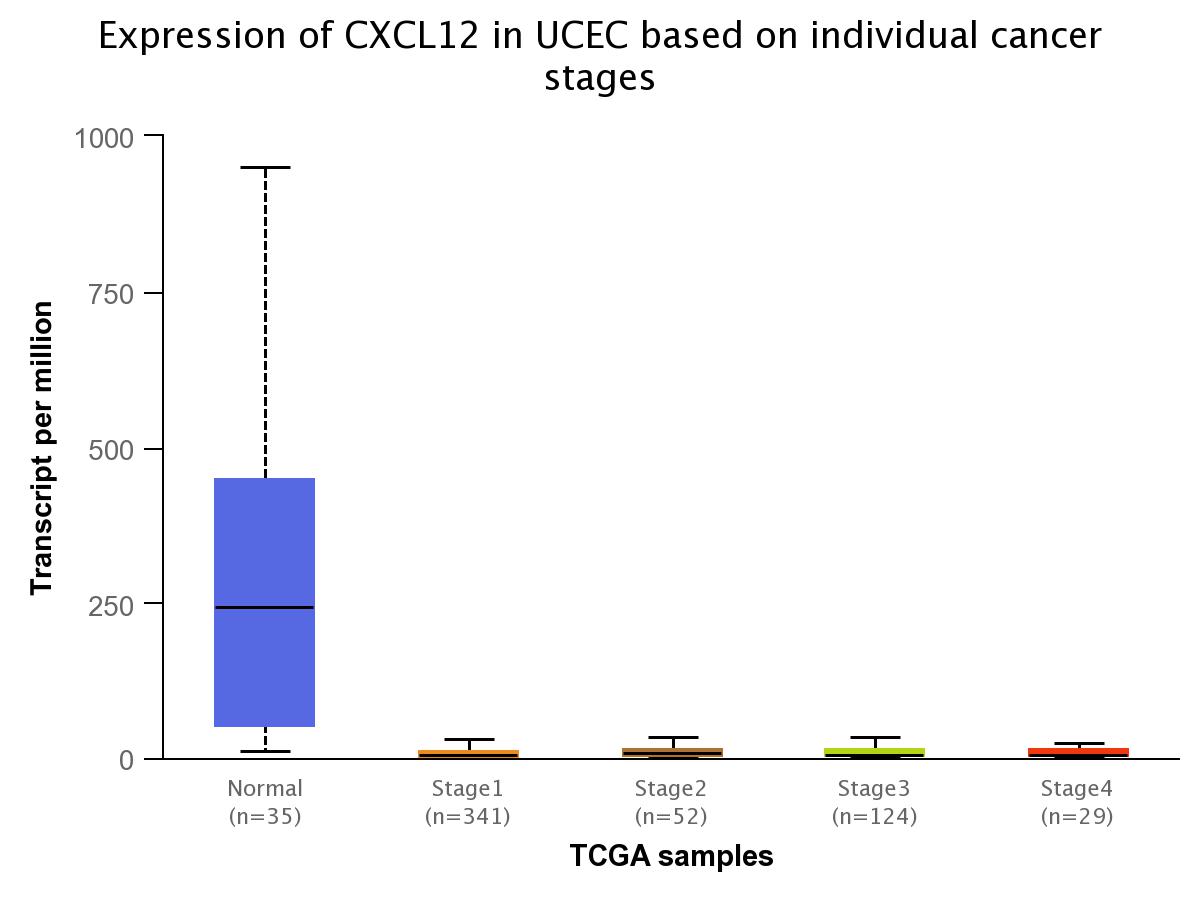

Supplement: S2 File — (ZIP) [file pone.0277872.s002.zip › S2_File/Figure 2B.jpeg]

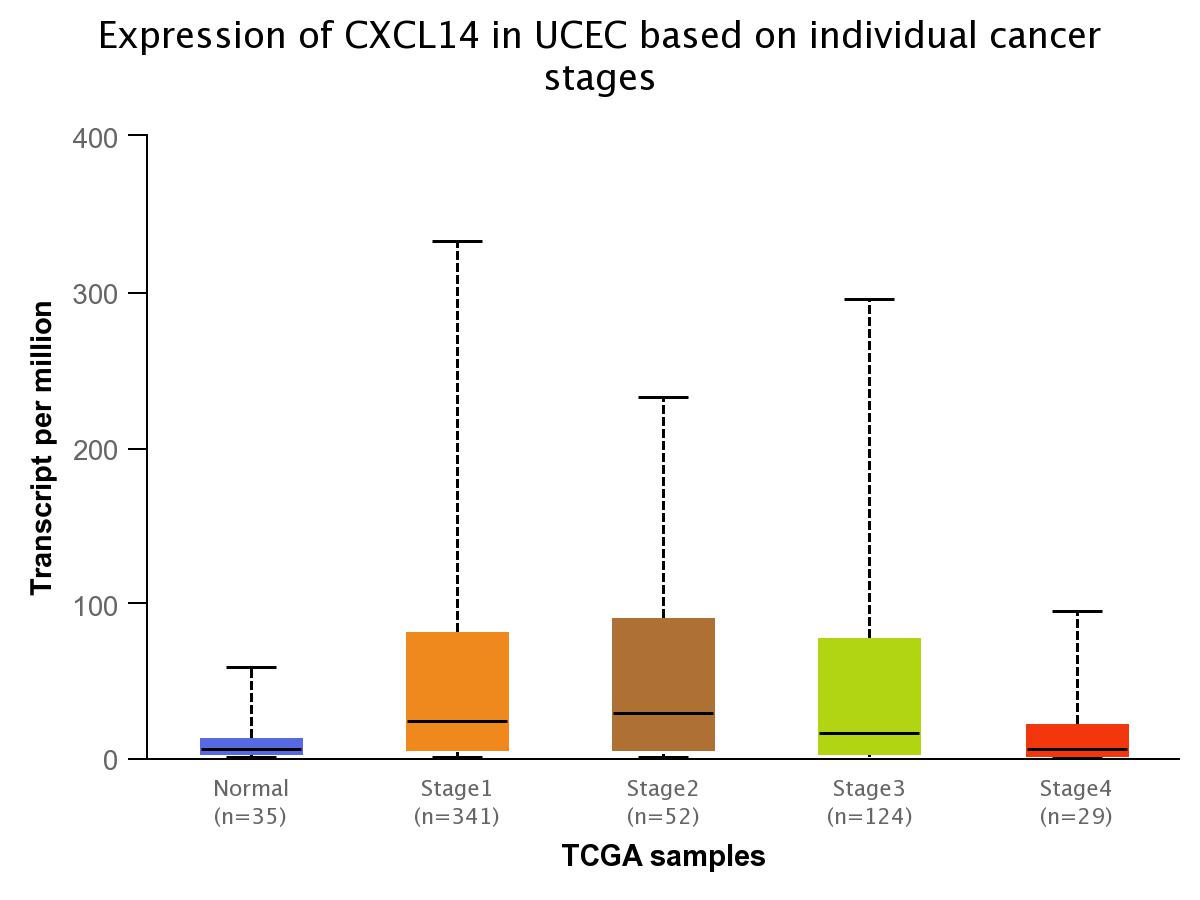

Supplement: S2 File — (ZIP) [file pone.0277872.s002.zip › S2_File/Figure 2C.jpeg]

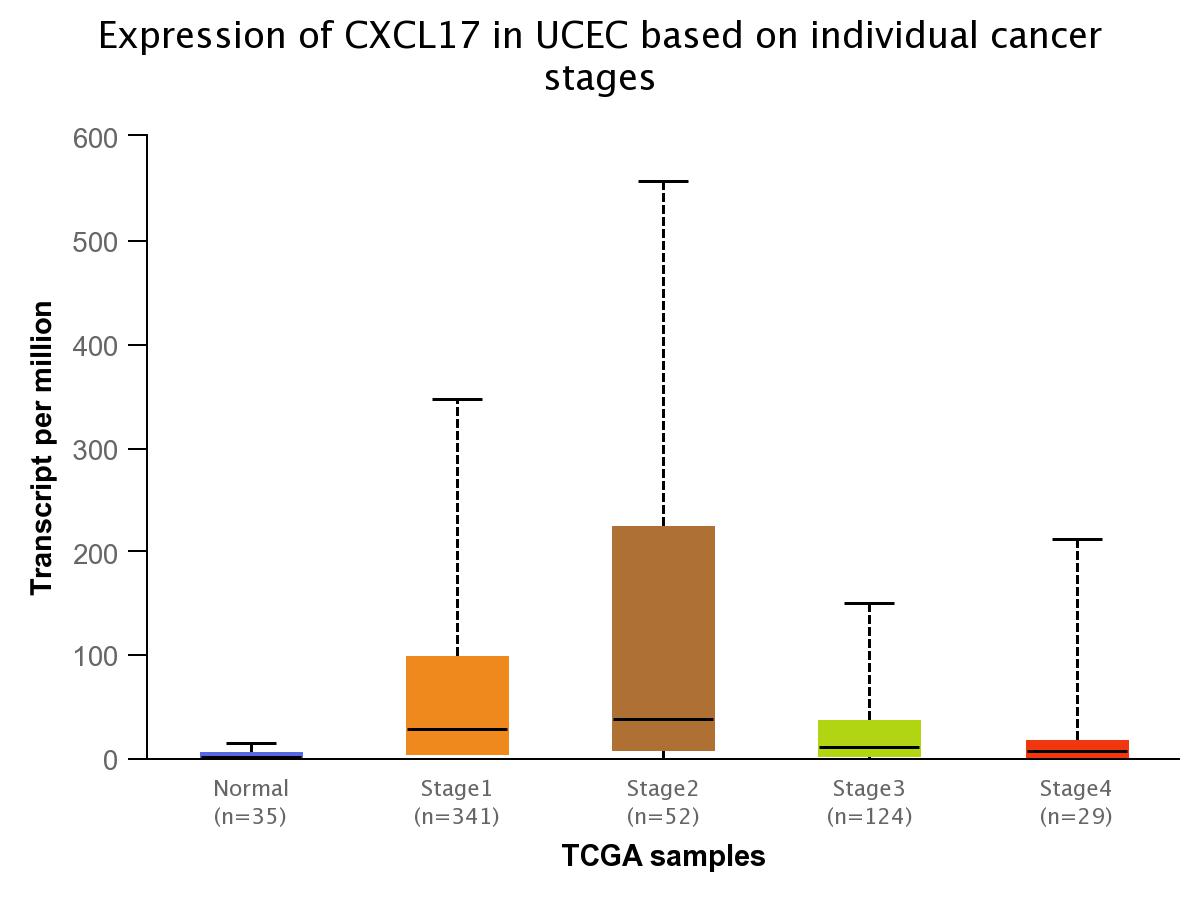

Supplement: S2 File — (ZIP) [file pone.0277872.s002.zip › S2_File/Figure 2D.jpeg]

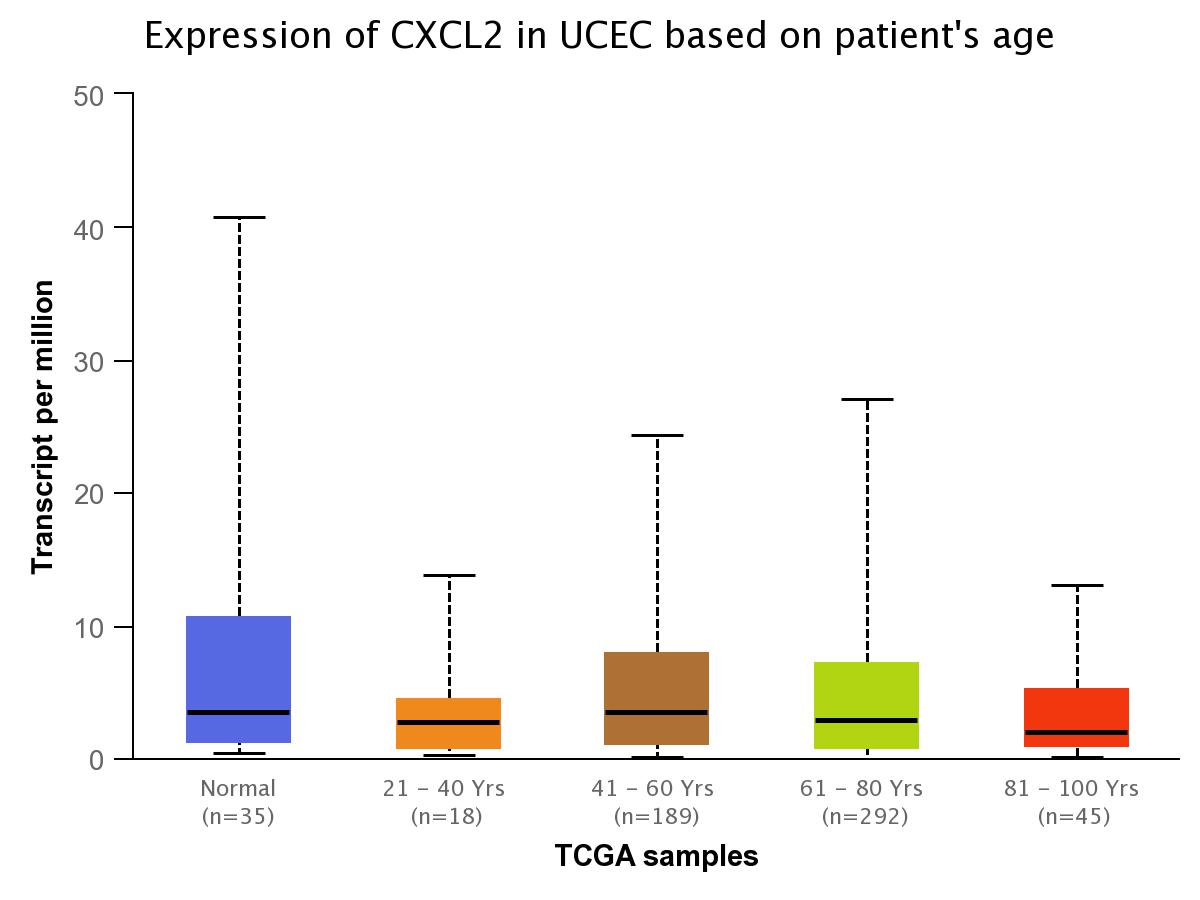

Supplement: S2 File — (ZIP) [file pone.0277872.s002.zip › S2_File/Figure 2E.jpeg]

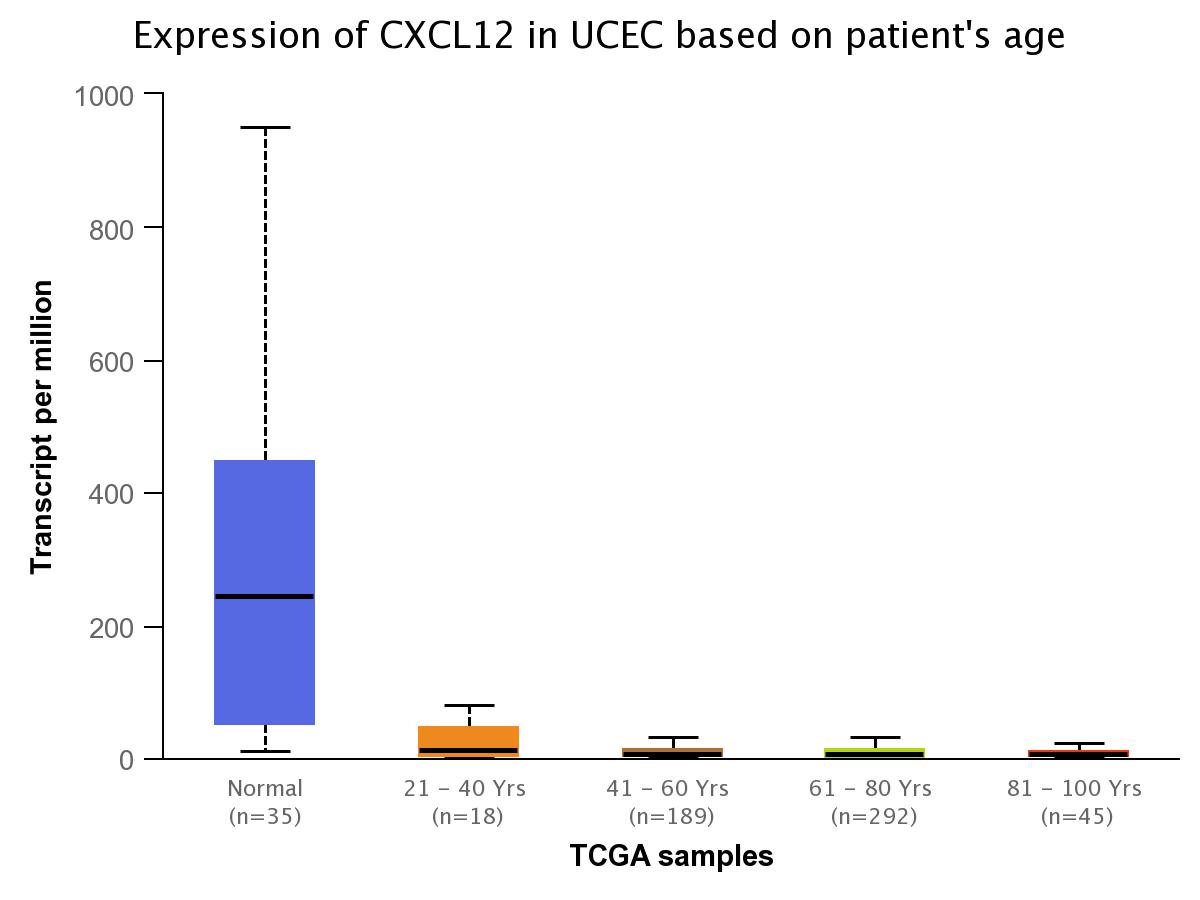

Supplement: S2 File — (ZIP) [file pone.0277872.s002.zip › S2_File/Figure 2F.jpeg]

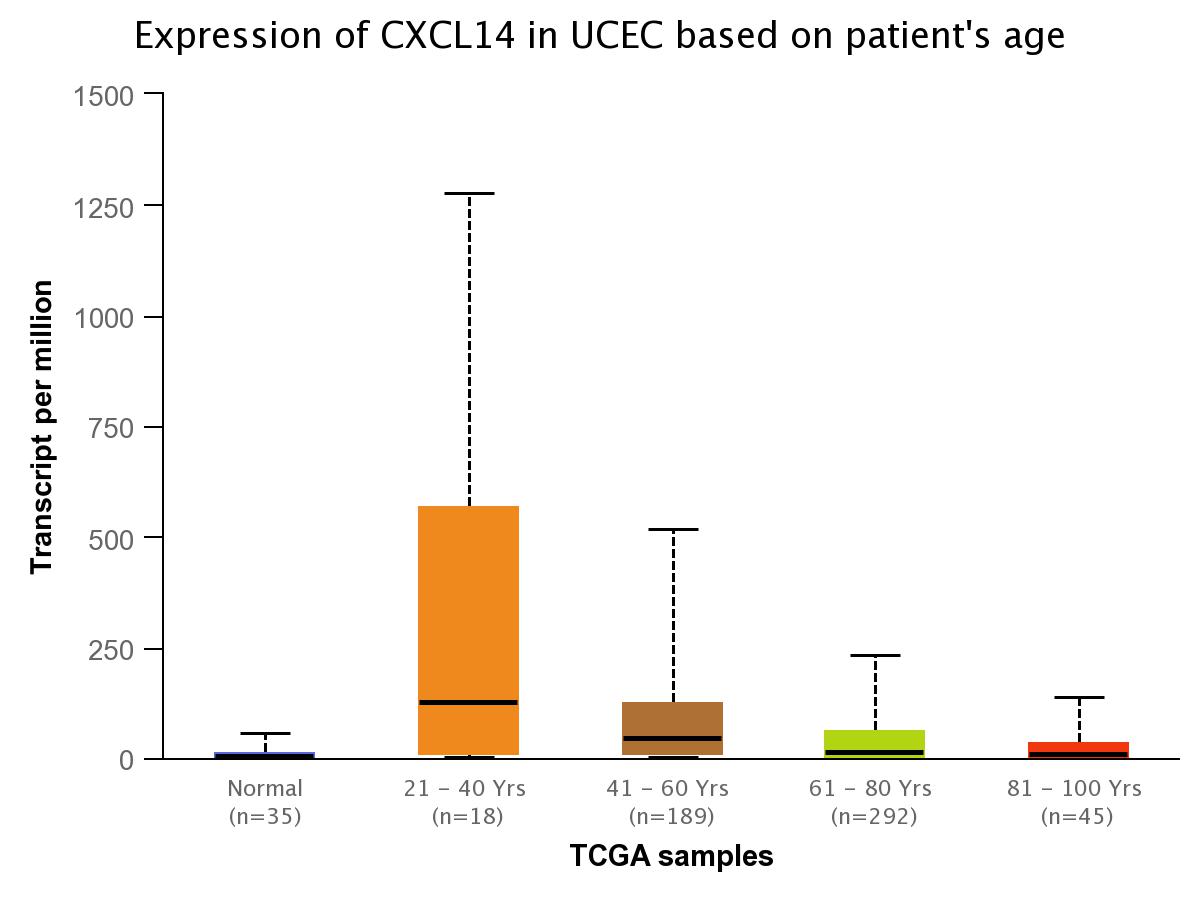

Supplement: S2 File — (ZIP) [file pone.0277872.s002.zip › S2_File/Figure 2G.jpeg]

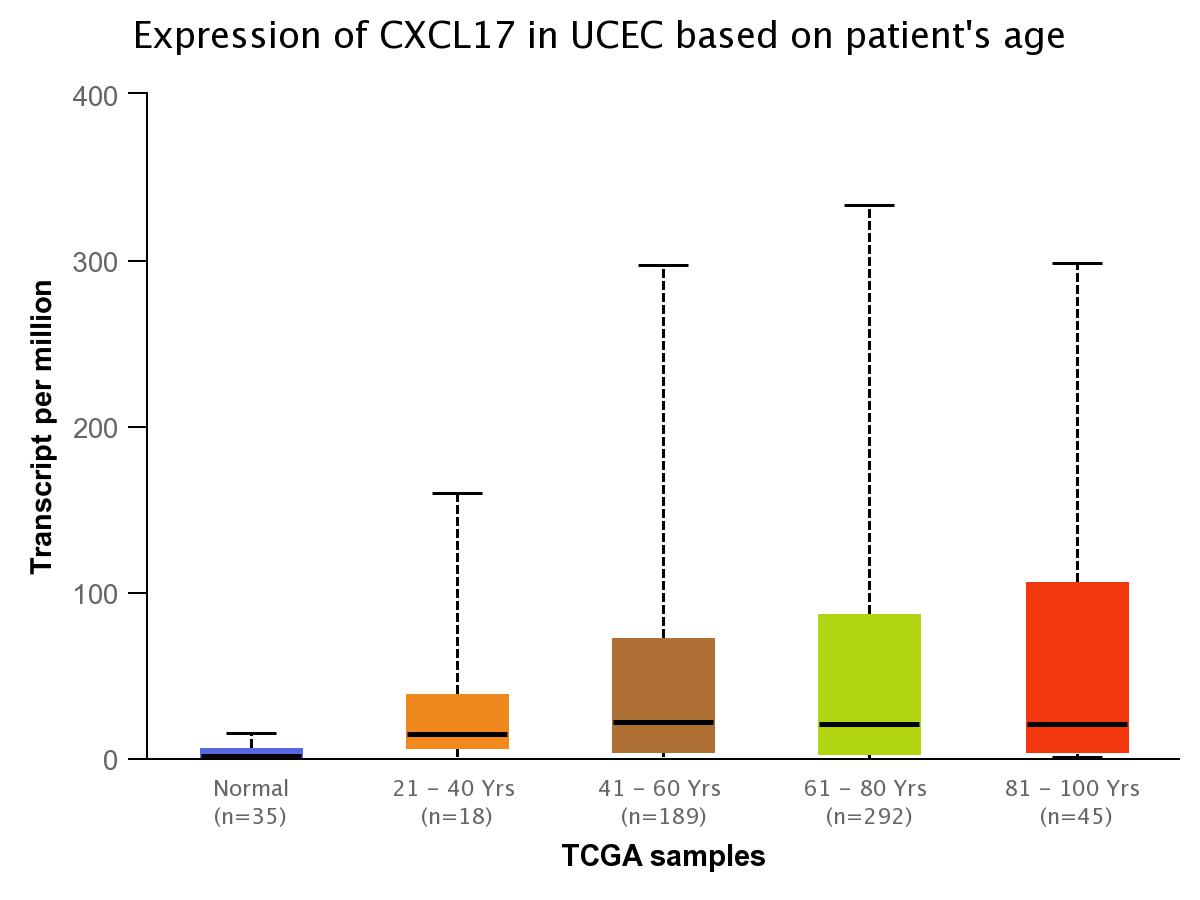

Supplement: S2 File — (ZIP) [file pone.0277872.s002.zip › S2_File/Figure 2H.jpeg]

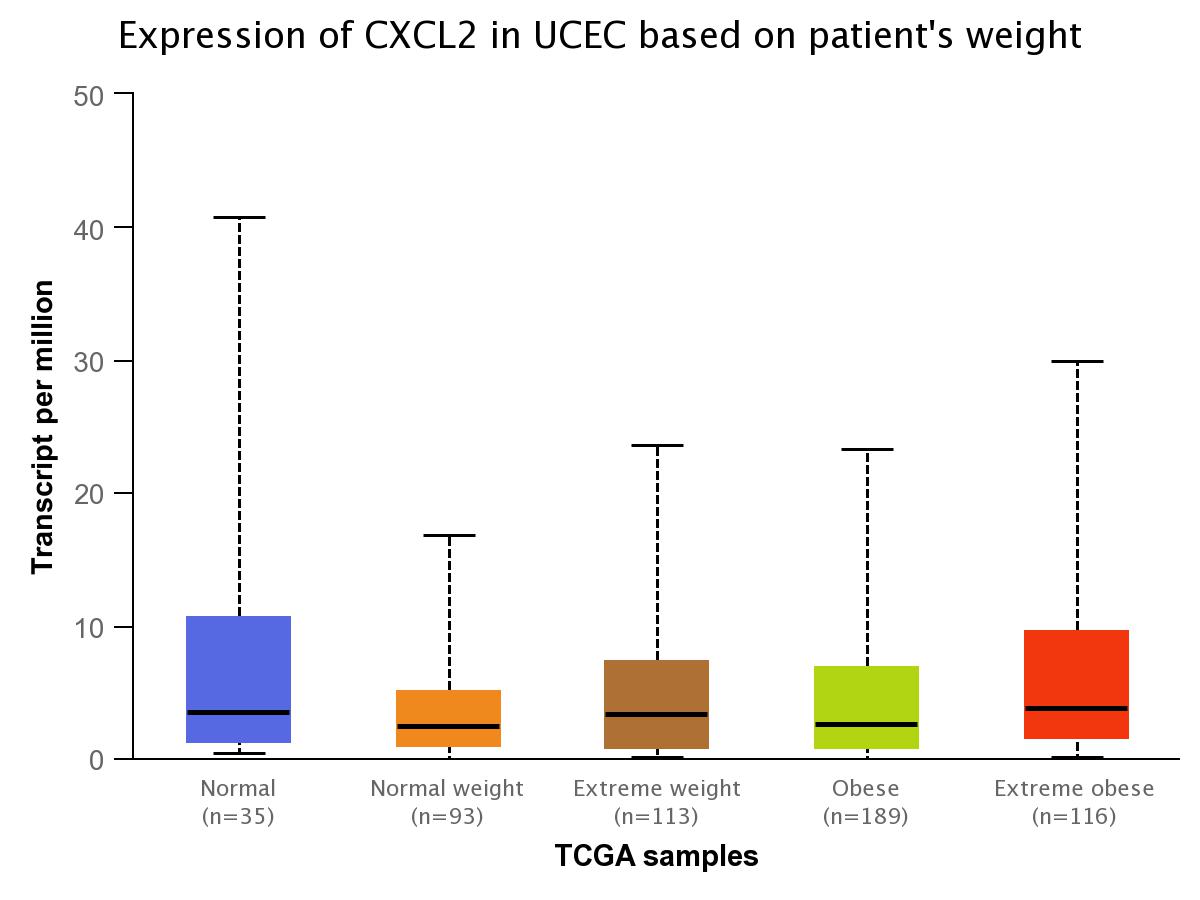

Supplement: S2 File — (ZIP) [file pone.0277872.s002.zip › S2_File/Figure 2I.jpeg]

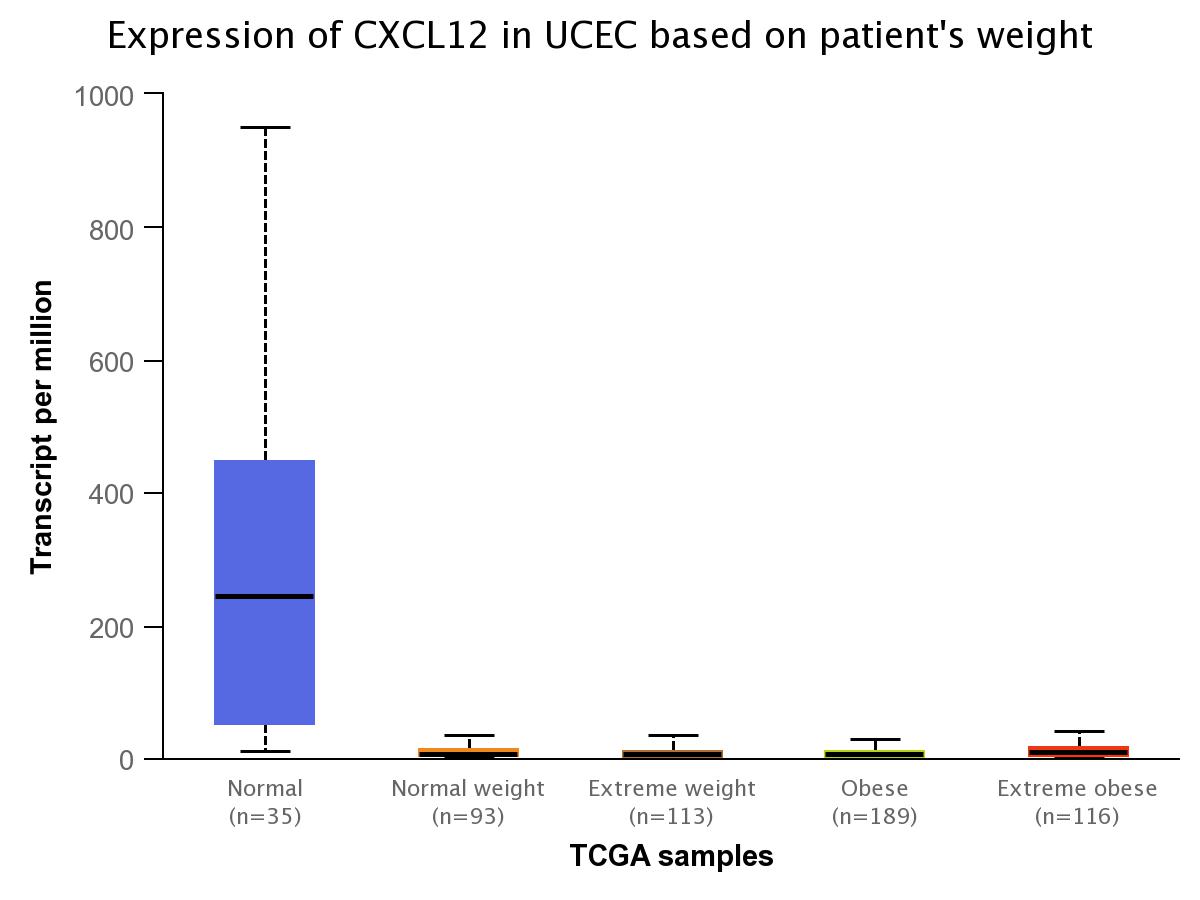

Supplement: S2 File — (ZIP) [file pone.0277872.s002.zip › S2_File/Figure 2J.jpeg]

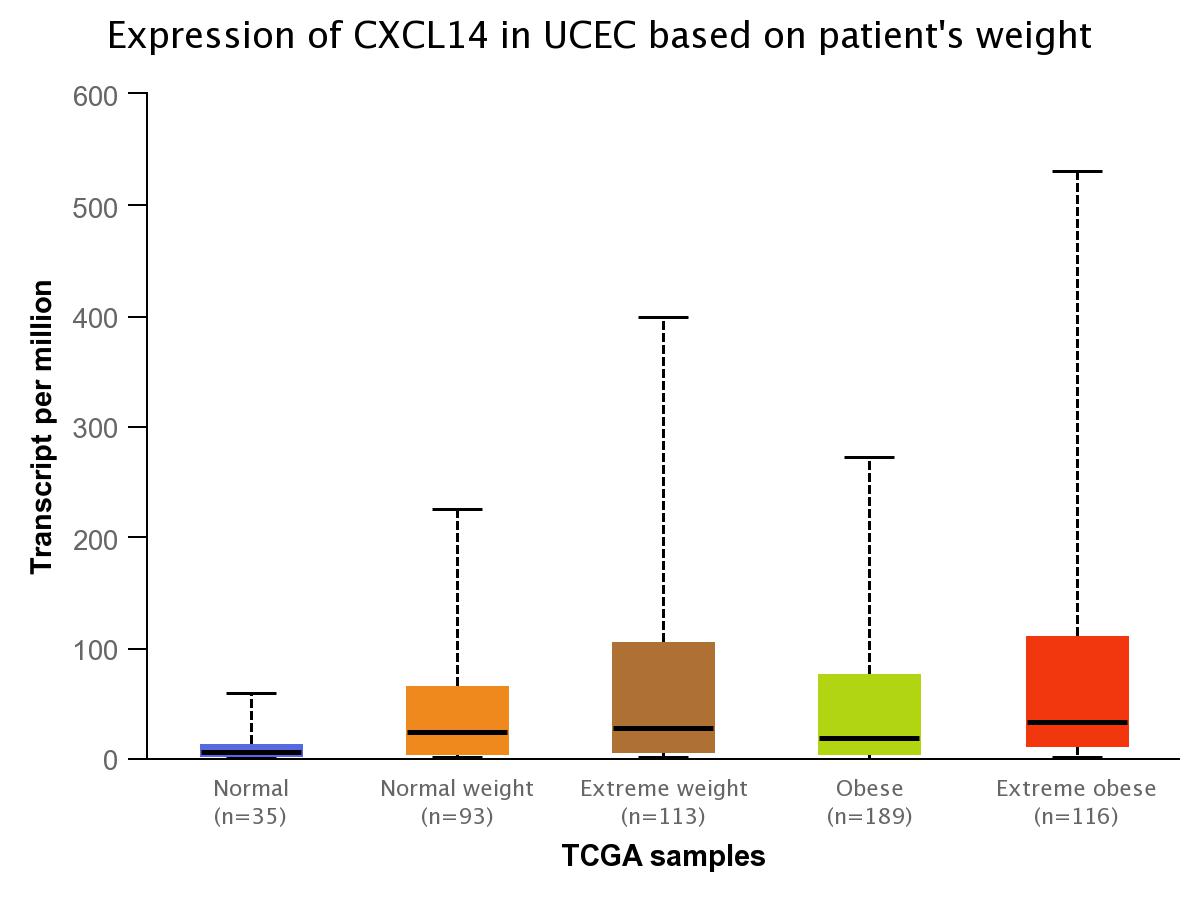

Supplement: S2 File — (ZIP) [file pone.0277872.s002.zip › S2_File/Figure 2K.jpeg]

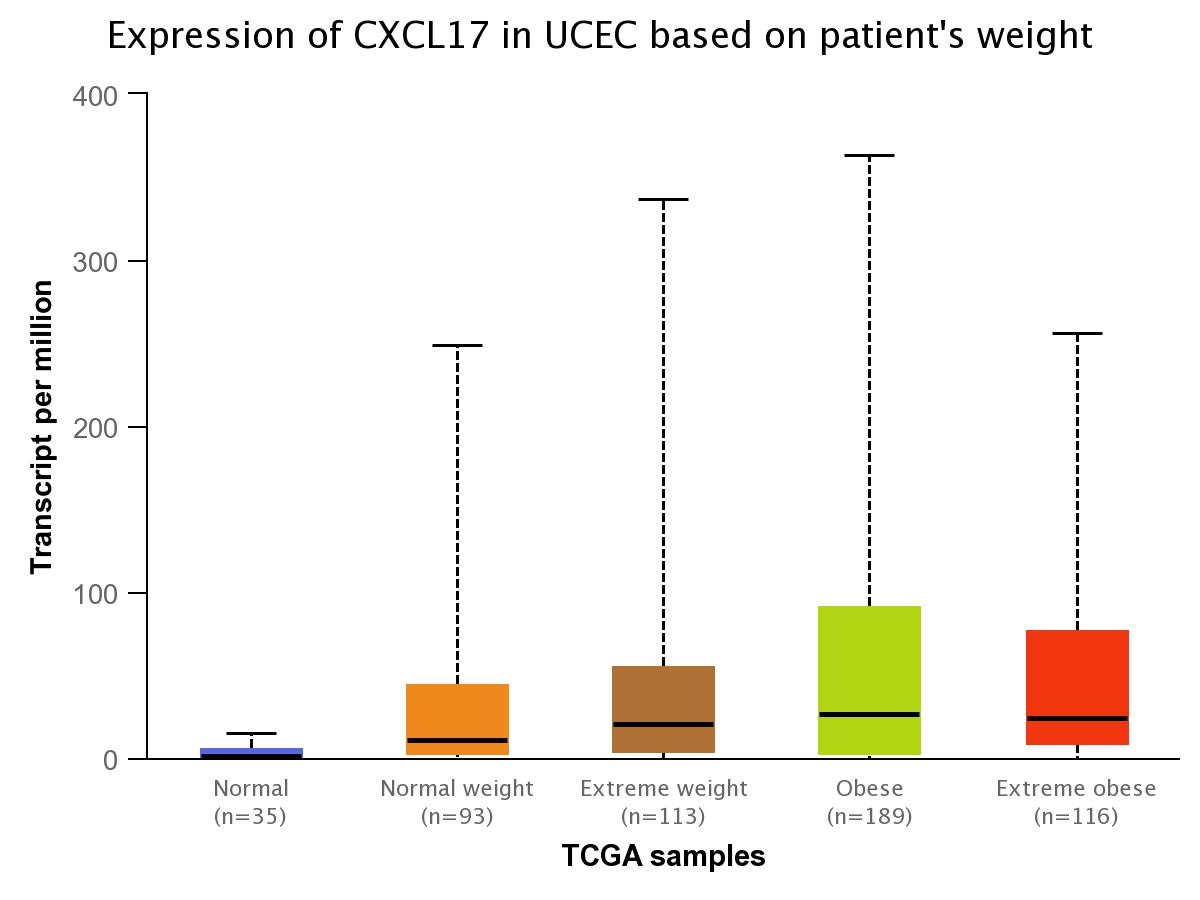

Supplement: S2 File — (ZIP) [file pone.0277872.s002.zip › S2_File/Figure 2L.jpeg]

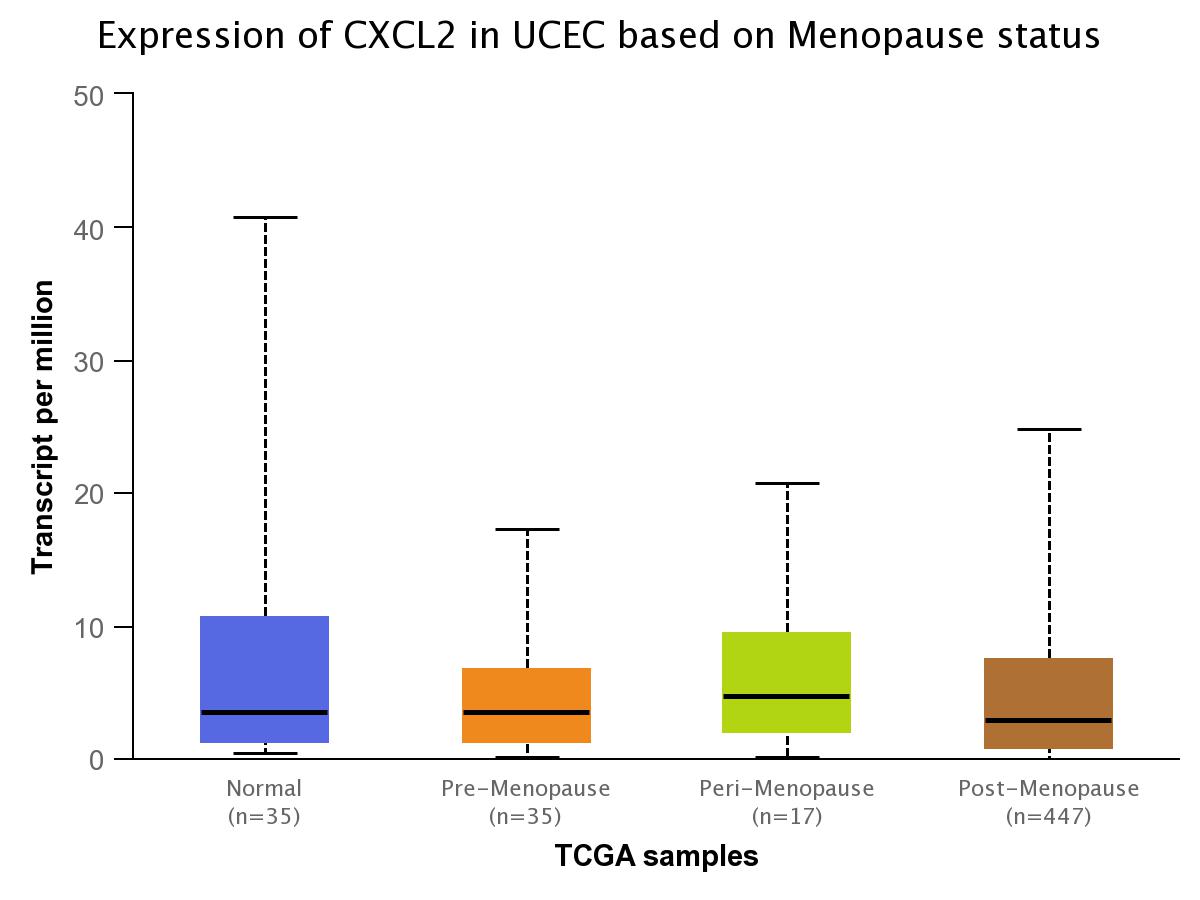

Supplement: S2 File — (ZIP) [file pone.0277872.s002.zip › S2_File/Figure 2M.jpeg]

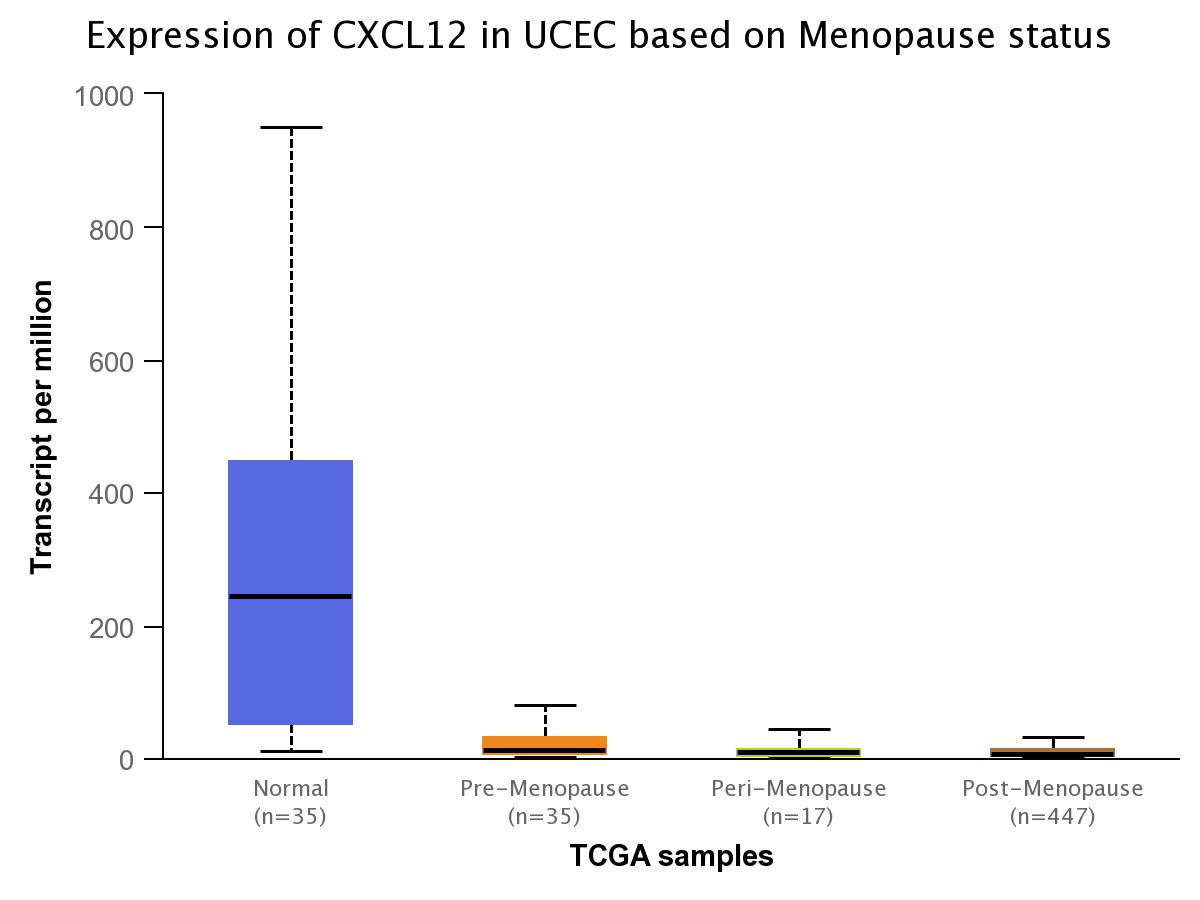

Supplement: S2 File — (ZIP) [file pone.0277872.s002.zip › S2_File/Figure 2N.jpeg]

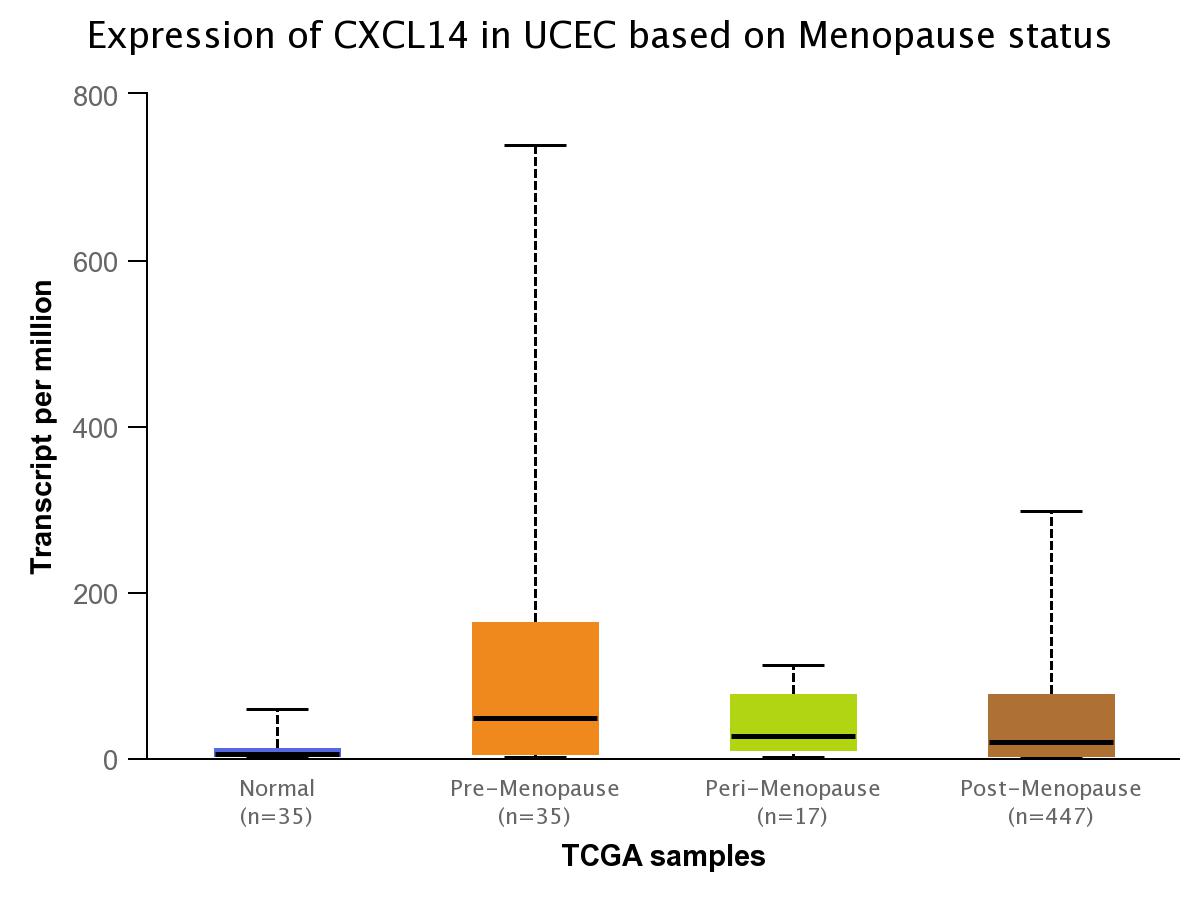

Supplement: S2 File — (ZIP) [file pone.0277872.s002.zip › S2_File/Figure 2O.jpeg]

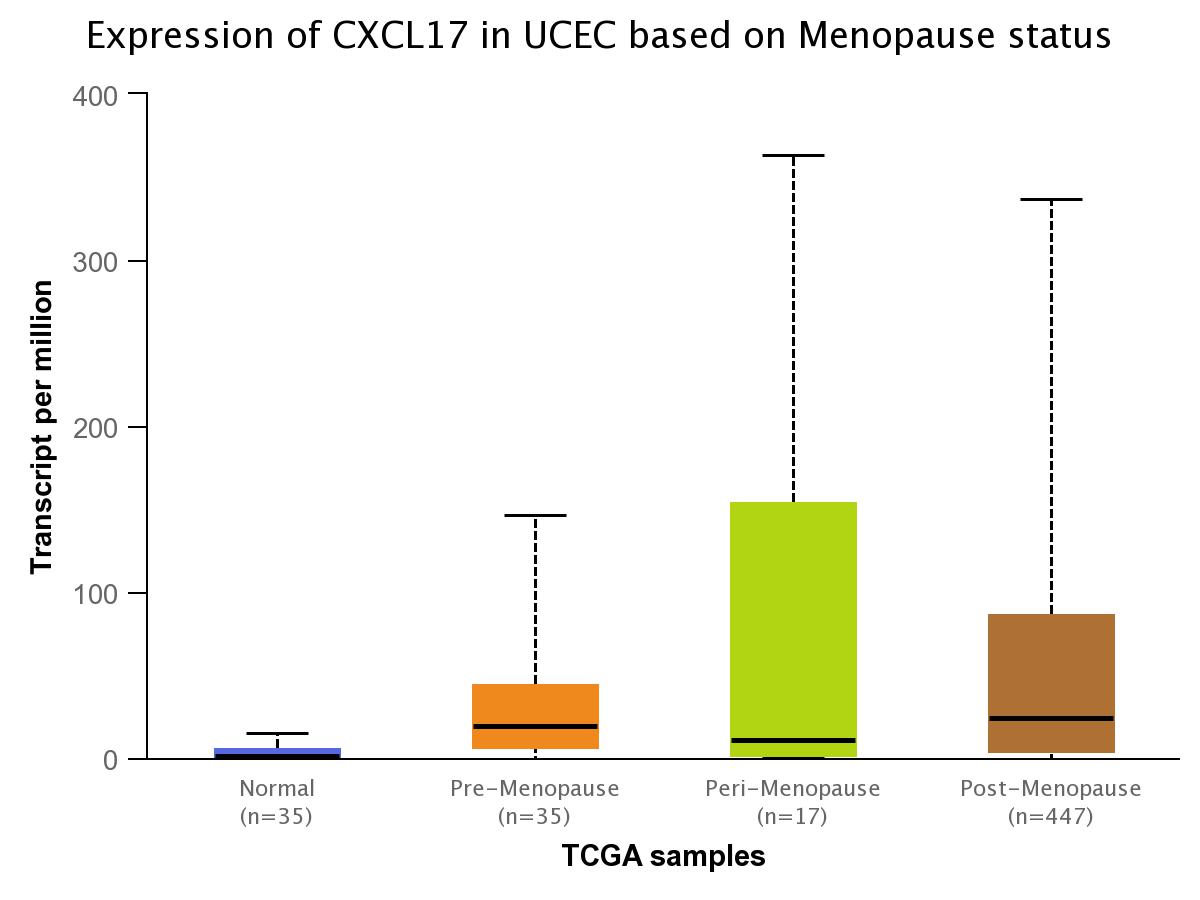

Supplement: S2 File — (ZIP) [file pone.0277872.s002.zip › S2_File/Figure 2P.jpeg]

# CXCL2

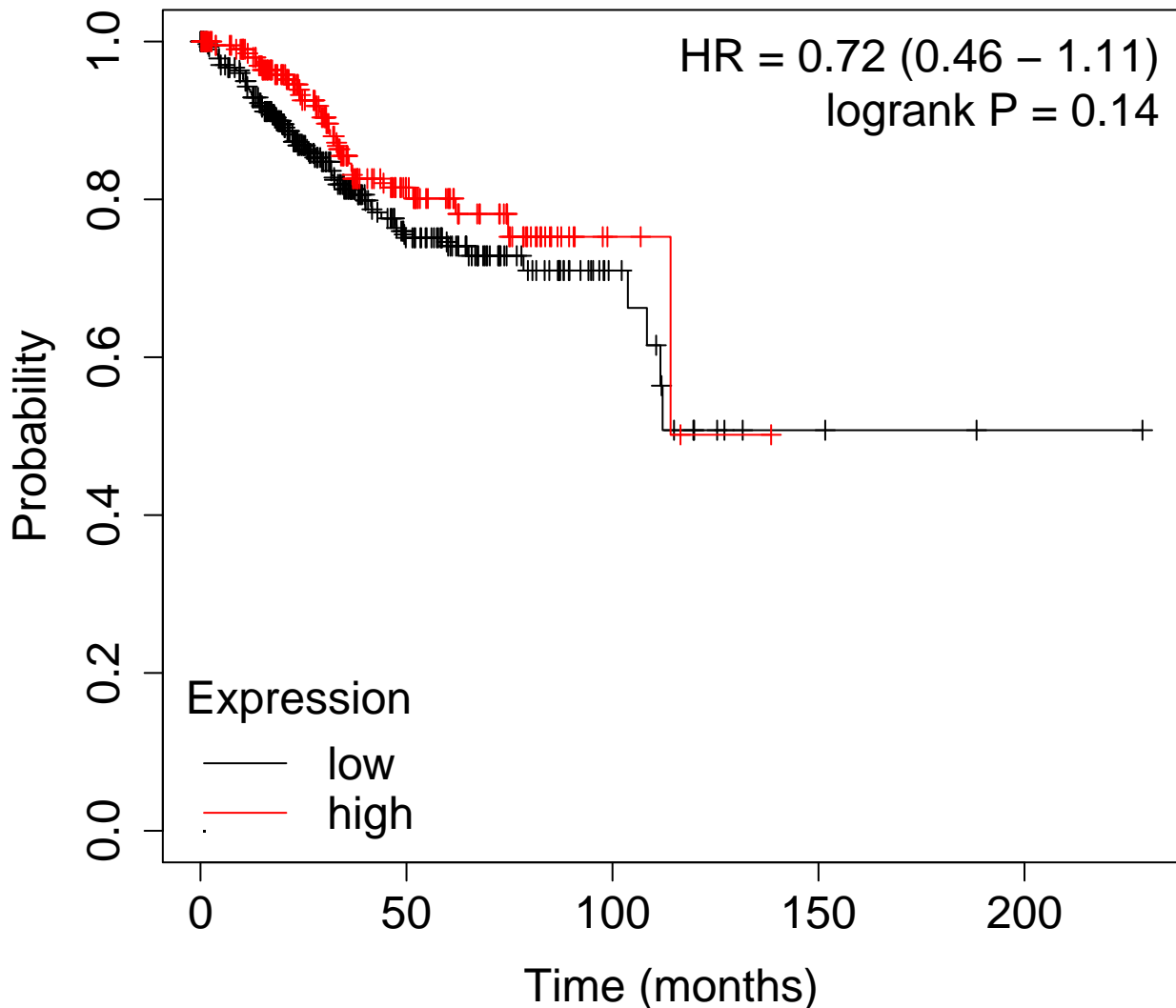

Number at risk

|      |     |    |    |   |   |
|------|-----|----|----|---|---|
| low  | 321 | 87 | 16 | 3 | 1 |
| high | 221 | 60 | 4  | 0 | 0 |

Supplement: S3 File — (ZIP) [file pone.0277872.s003.zip › S3_File/Figure 3A.pdf]

# CXCL12

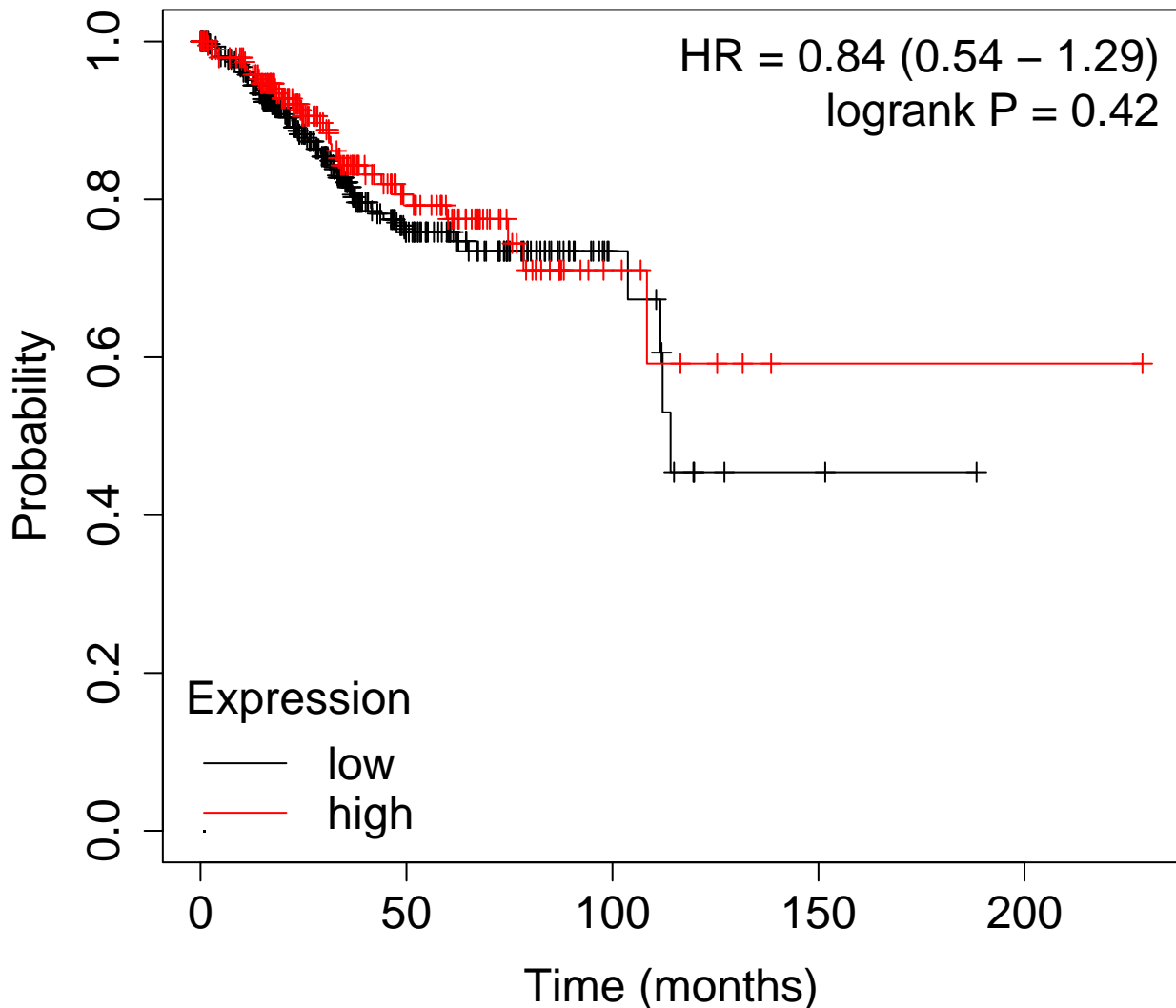

Number at risk

|      |     |    |    |   |   |
|------|-----|----|----|---|---|
| low  | 335 | 89 | 12 | 2 | 0 |
| high | 207 | 58 | 8  | 1 | 1 |

Supplement: S3 File — (ZIP) [file pone.0277872.s003.zip › S3_File/Figure 3B.pdf]

# CXCL14

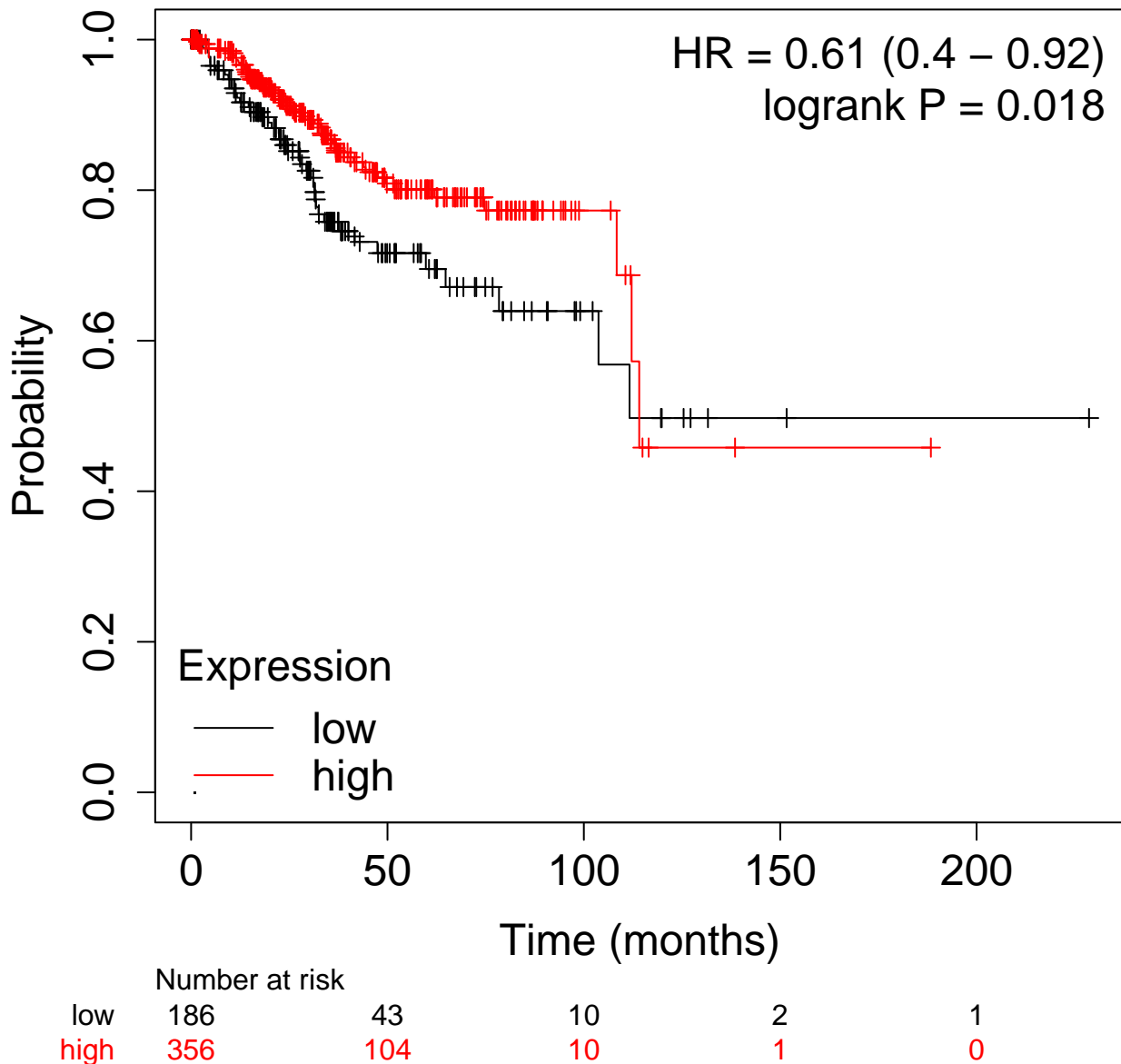

Supplement: S3 File — (ZIP) [file pone.0277872.s003.zip › S3_File/Figure 3C.pdf]

# CXCL17

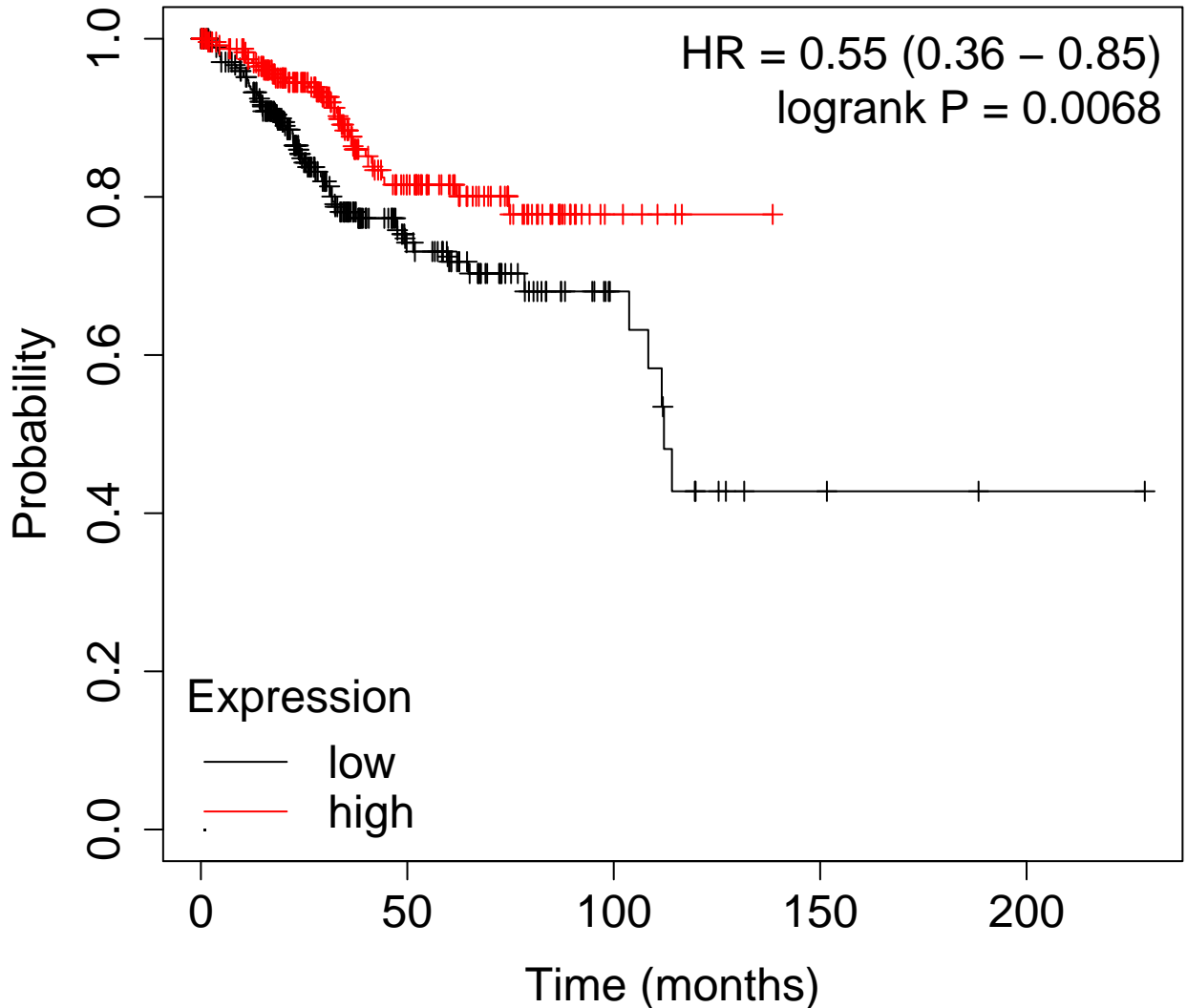

Number at risk

|      |     |    |    |   |   |
|------|-----|----|----|---|---|
| low  | 288 | 67 | 14 | 3 | 1 |
| high | 254 | 80 | 6  | 0 | 0 |

Supplement: S3 File — (ZIP) [file pone.0277872.s003.zip › S3_File/Figure 3D.pdf]

# CXCL2

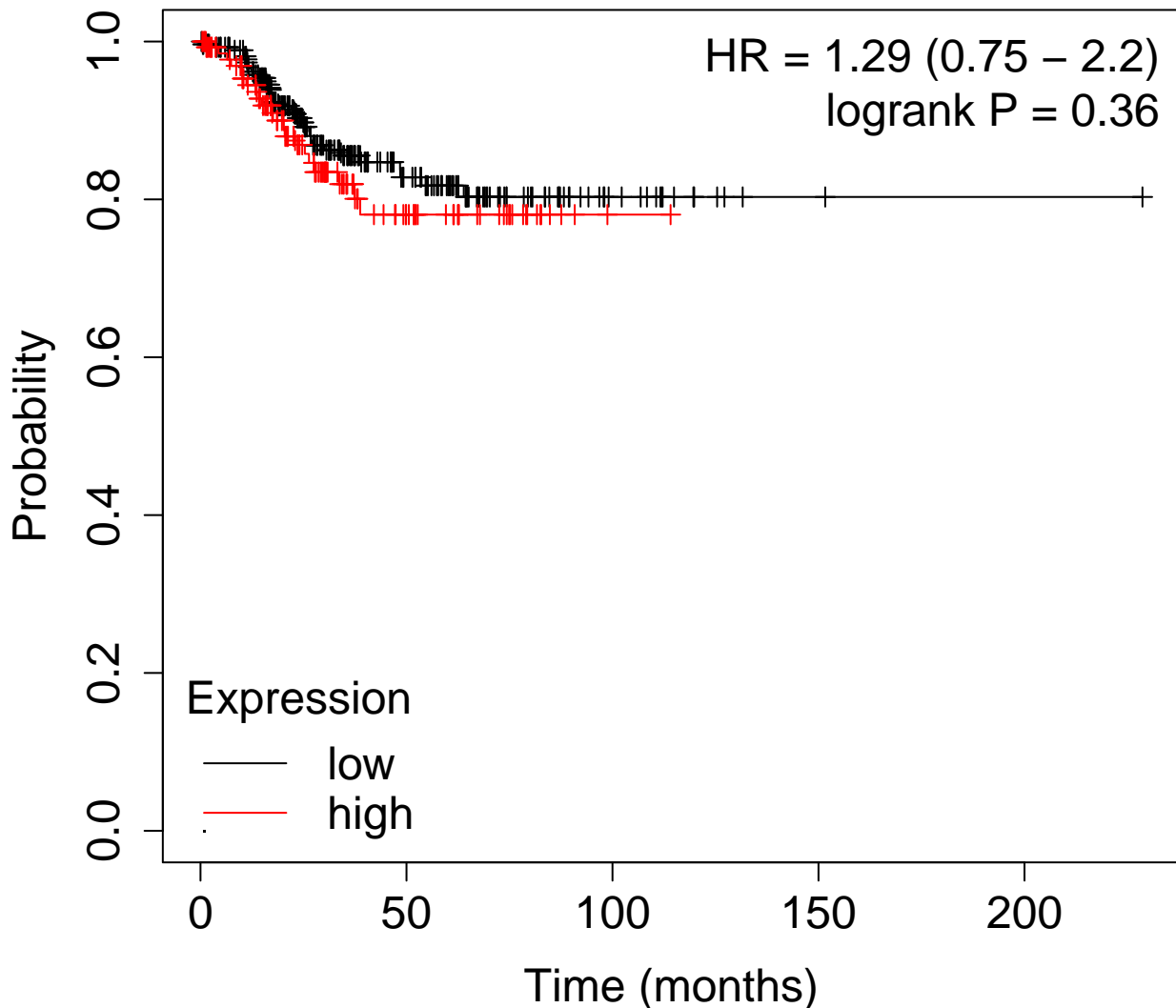

Number at risk

|      |     |    |    |   |   |
|------|-----|----|----|---|---|
| low  | 280 | 83 | 15 | 2 | 1 |
| high | 142 | 32 | 1  | 0 | 0 |

Supplement: S3 File — (ZIP) [file pone.0277872.s003.zip › S3_File/Figure 3E.pdf]

# CXCL12

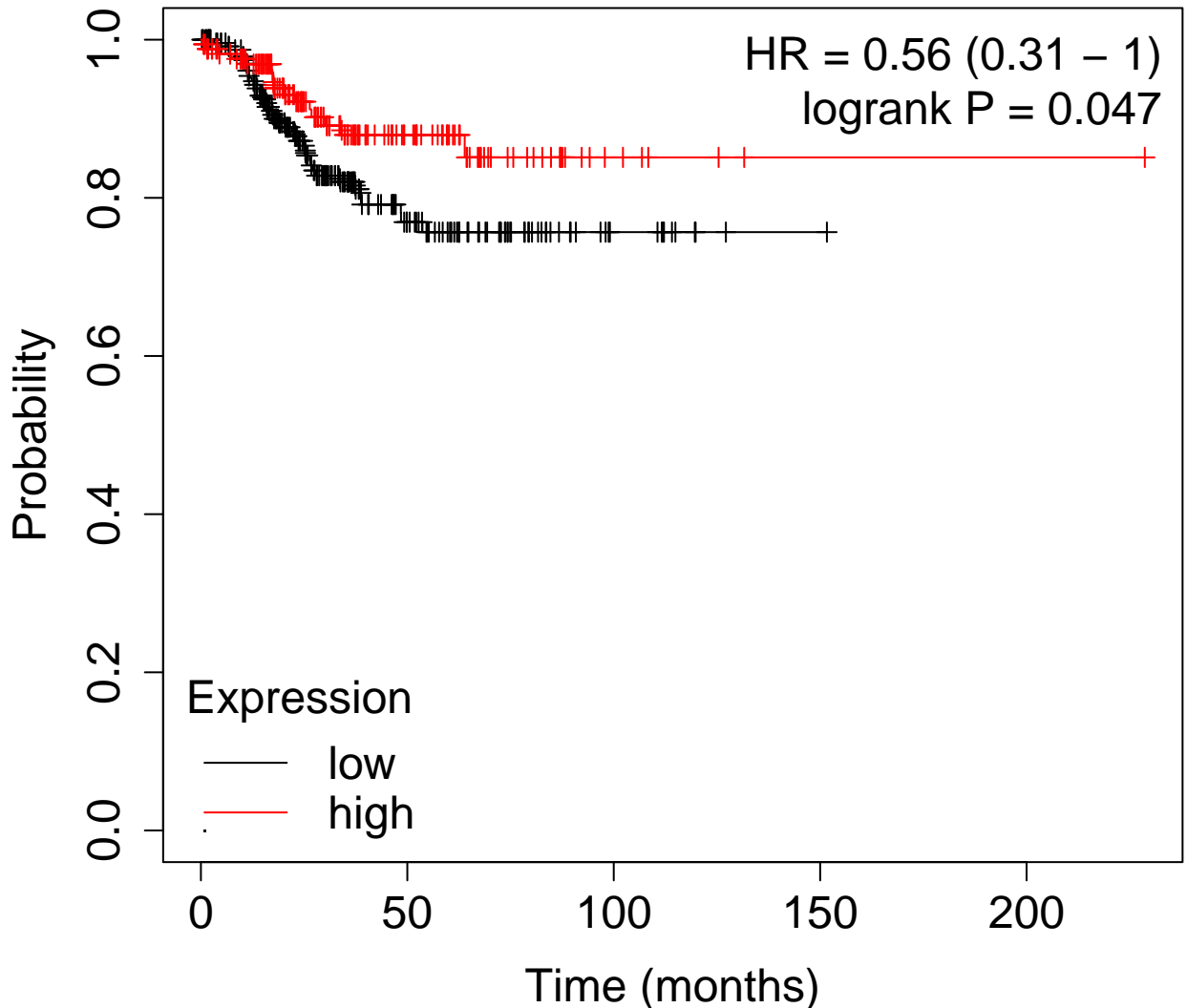

Number at risk

|      |     |    |    |   |   |
|------|-----|----|----|---|---|
| low  | 251 | 66 | 10 | 1 | 0 |
| high | 171 | 49 | 6  | 1 | 1 |

Supplement: S3 File — (ZIP) [file pone.0277872.s003.zip › S3_File/Figure 3F.pdf]

# CXCL14

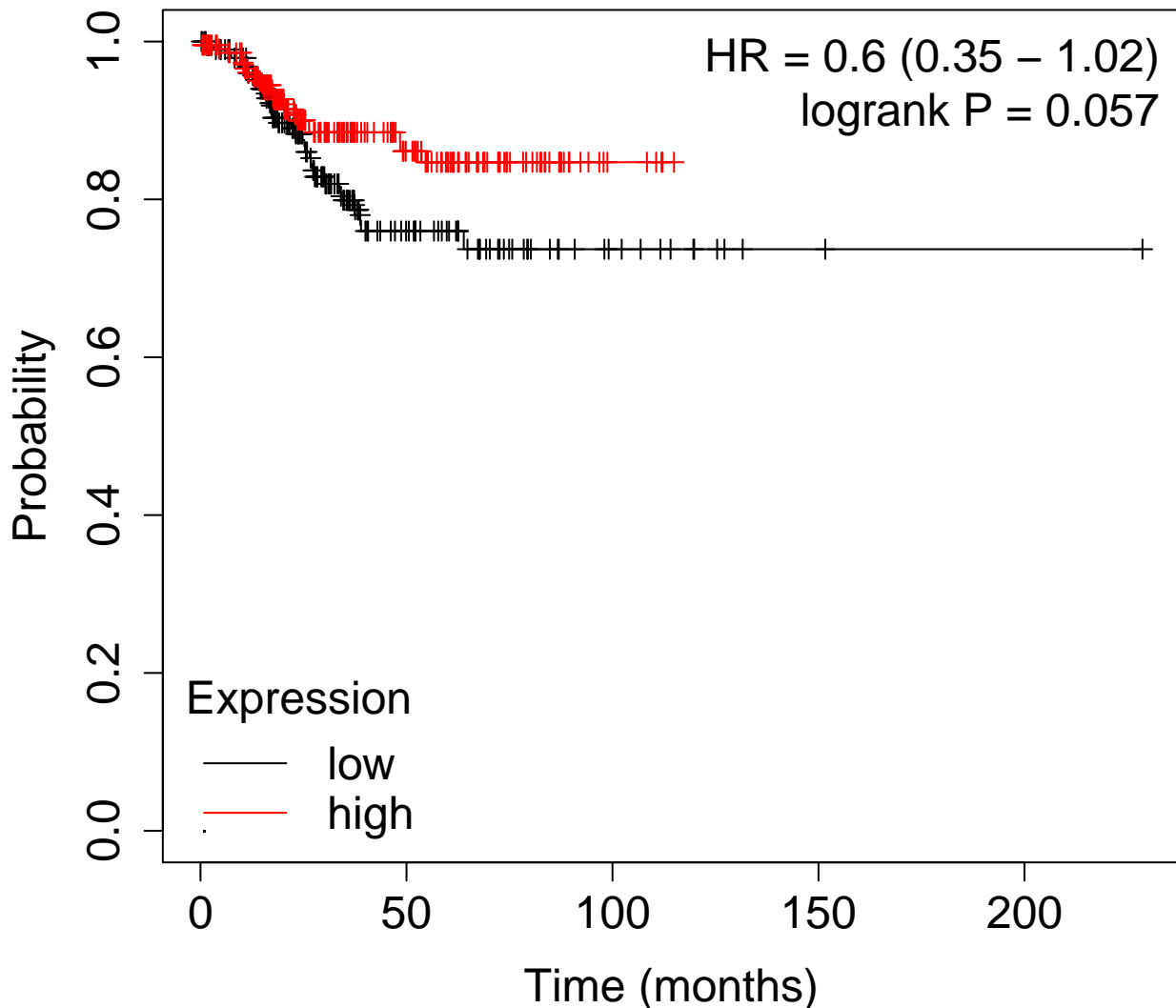

| Number at risk |     |    |    |   |   |
|----------------|-----|----|----|---|---|
| low            | 202 | 47 | 11 | 2 | 1 |
| high           | 220 | 68 | 5  | 0 | 0 |

Supplement: S3 File — (ZIP) [file pone.0277872.s003.zip › S3_File/Figure 3G.pdf]

# CXCL17

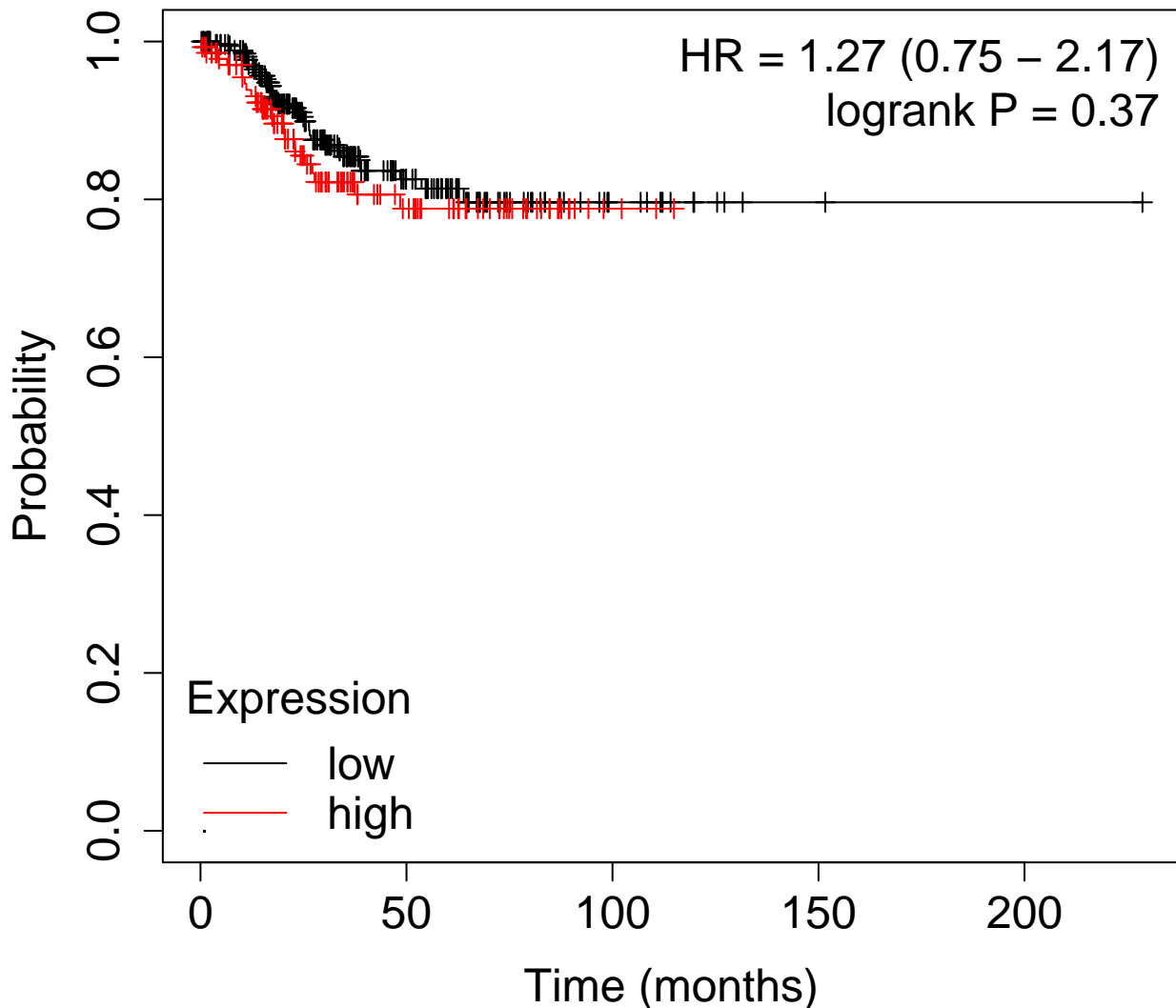

Number at risk

|      |     |    |    |   |   |
|------|-----|----|----|---|---|
| low  | 282 | 72 | 13 | 2 | 1 |
| high | 140 | 43 | 3  | 0 | 0 |

Supplement: S3 File — (ZIP) [file pone.0277872.s003.zip › S3_File/Figure 3H.pdf]
